# Reprogramming Immunogenicity of Iron Oxide Nanoparticles through Sulfated Glycan Presentation

**DOI:** 10.1002/smll.202508613

**Published:** 2026-02-01

**Authors:** Negin Pournoori, Heela Sarlus, Dick J. Sjöström, Rohith Pavan Parvathaneni, Oommen P. Varghese, Vesa P. Hytönen, Robert A. Harris, Per H. Nilsson, Oommen P. Oommen

**Affiliations:** ^1^ Faculty of Medicine and Health Technology Tampere University Tampere Finland; ^2^ Department of Clinical Neuroscience Karolinska Institutet, Centre for Molecular Medicine, Karolinska Hospital Sweden; ^3^ Linnaeus Centre for Biomaterials Chemistry Linnaeus University Kalmar Sweden; ^4^ Department of Chemistry and Biomedicine Linnaeus University Kalmar Sweden; ^5^ Department of Chemistry‐Ångström Laboratory, Translational Chemical Biology Group, Division of Macromolecular Chemistry Uppsala University Uppsala Sweden; ^6^ Fimlab Laboratories Tampere Finland; ^7^ School of Pharmacy and Pharmaceutical Sciences Cardiff University Cardiff UK

**Keywords:** dextran sulfate, heparin, immunomodulatory effects, macrophage polarization, superparamagnetic iron oxide nanoparticles

## Abstract

Heparin (HP) and dextran sulfate (DS) are well‐known for their anti‐thrombotic and immunomodulatory properties; however, a direct comparison of their immunological responses when used in drug delivery applications is lacking. This study addresses this gap by evaluating the immunological behavior of superparamagnetic iron oxide nanoparticles (SPIONs) coated with HP or DS in human whole blood, primary immune cells, endothelial cells, and in vivo. Both HP‐SPIONs and DS‐SPIONs effectively suppressed complement activation, as shown by reduced C3bc, C3bBbP, and TCC levels. Notably, HP‐SPIONs activated monocytes (CD11b) and endothelial cells (ICAM‐1, CD62P/E), whereas DS‐SPIONs suppressed endothelial activation. DS‐SPIONs were preferentially internalized by myeloid cells (∼50% neutrophils, ∼42% macrophages, ∼55% dendritic cells), while HP‐SPIONs showed significantly lower uptake (<25% dendritic cells, ∼5% neutrophils). DS‐SPIONs induced an immunosuppressive, pro‐healing phenotype in murine and human macrophages, whereas HP‐SPIONs drove a pro‐inflammatory, M1‐like response. In healthy mice, intravenous DS‐SPIONs elicited a modest increase in splenic immune cell populations compared to HP‐SPIONs, indicating early immune engagement. Collectively, both SPIONs attenuate complement activation, indicating high biocompatibility. Based on the early immunological responses, DS‐SPIONs display a pro‐healing immune profile suitable for regenerative drug delivery, whereas HP‐SPIONs induce pro‐inflammatory responses that may be leveraged for anticancer immunotherapy.

## Graphical abstract

This study presents a comparative analysis of heparin (HP)‐ and dextran sulfate (DS)‐coated superparamagnetic iron oxide nanoparticles (SPIONs) as a tunable immunomodulatory platform where surface chemistry dictates therapeutic function. DS‐SPIONs are preferentially internalized by myeloid cells, reprogramming them into a pro‐healing M2 phenotype, while HP‐SPIONs drive a pro‐inflammatory M1 response. By simply altering the polysaccharide coating, this platform achieves divergent macrophage polarization, offering a rational design strategy for either anti‐cancer immunotherapy or regenerative medicine.

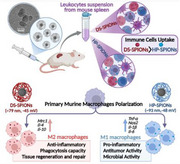

## 1 Introduction

Biopolymers in the extracellular matrix such as glycosaminoglycans act as bioactive interfaces that bind and present cytokines and engage immune cells, modulating the onset, amplitude, and resolution of inflammatory responses [1]. Heparin (HP) is a highly sulfated glycosaminoglycan with well‐documented immunomodulatory and antithrombotic properties. HP is clinically tested for treating various thrombotic conditions and inflammatory diseases, namely reactive airway disease and asthma, inflammatory bowel disease, acute coronary syndrome and ischemic cerebrovascular events [2, 3]. Of particular interest are its anti‐cancer [4] and anti‐metastatic properties [5], which are mediated by its ability to suppress heparinase activity, regulate inflammation, and remodel the tumor microenvironment by inhibiting angiogenesis and blocking P‐selectin [6]. Despite its outstanding bioactivity, however, the clinical use of HP, especially for anticancer applications, is severely limited by complications such as bleeding risks and heparin‐induced thrombocytopenia [3, 7]. This creates a pressing need for an alternative that retains the therapeutic immunological and anti‐tumor activities of HP while mitigating its adverse effects. Dextran sulfate (DS) is a promising alternative candidate as it exhibits several bioactivities analogous to HP [8], including potent complement inhibition [9] and anti‐metastatic activity [10]. Crucially, DS possesses a distinct and therapeutically valuable immunomodulatory profile. It is effectively employed to target activated macrophages via scavenger receptors, a strategy that has been evaluated in preclinical models of rheumatoid arthritis and atherosclerosis [8, 11, 12]. Furthermore, its immunoprotected qualities have led to its use as a cytoprotectant in solid organ and islet transplantation [13]. Notably, low molecular weight DS mitigates instant blood‐mediated inflammatory reactions (IBMIR) more efficiently than heparin, a significant advantage that has been harnessed in clinical trials for pancreatic islet transplantation [14, 15].

Despite the promising biological properties of both HP and DS for drug delivery and immunomodulation, a direct comparative study for drug delivery applications is currently lacking. To address this gap, we engineered HP‐ and DS‐coated superparamagnetic iron oxide nanoparticles (SPIONs) as a multifunctional theranostic platform with significant translational potential in drug delivery, immunotherapy, and magnetic resonance imaging (MRI) [16, 17]. SPIONs exhibit well‐defined and tunable immune interactions, where their size, surface charge, and coating chemistry dictate complement activation, cellular uptake, and macrophage polarization. Typically, uncoated or cationic SPIONs activate complement via the alternative pathway, whereas hydrophilic or anionic coatings mitigate C3 and C5 cleavage, resulting in controlled immune interactions and biodegradation into physiological iron metabolism [18, 19]. This inherent tunability provides an ideal experimental system to investigate how distinct polysaccharide coatings differentially modulate immune responses. Therefore, while both HP‐ and DS‐coated SPIONs have been independently explored for applications like MRI contrast enhancement or inflammation imaging, their differential impact on the immune system has not been systematically investigated [20, 21, 22].

In this study, we engineered SPIONs coated with either HP or DS as potential drug delivery platforms, and systematically and comparatively evaluated their immunomodulatory properties. This included assessment of complement activation in human whole blood, interactions with endothelial and immune cells, and cellular uptake by primary myeloid and lymphoid populations in the spleens of healthy mice as well as in primary immune cells. Our findings demonstrate that the surface coating of these nanocarriers profoundly influences their uptake by immune cells, activation of the innate immune system, endothelial activation, and macrophage polarization. These insights pave the way for designing precision nanomedicine strategies that target the immune system and that modulate multiple immune pathways to enhance the efficacy of drug delivery applications.

## 2 Results and Discussion

### 2.1 Synthesis and Characterization of Nanoparticle Systems

The bioactivity of nanoparticles, including SPIONs, is largely dictated by their surface coating, which influences their interaction with serum and complement proteins, thereby affecting biodistribution and clearance from circulation. This study assessed how HP and DS coating of SPIONs impacted immune modulation and cellular uptake by myeloid and lymphoid immune systems. Several different approaches were adopted to coat HP and DS on SPIONs. Conventionally, both unfractionated heparin (UFH) and dextran/dextran sulfate (DS)‐coated SPIONs are synthesized via a two‐step process. This typically involves i) the synthesis of bare SPIONs (Fe_3_O_4_) through the reaction of ferrous and ferric salts under alkaline conditions, followed by ii) the coating of these bare SPIONs with unmodified UFH or dextran/DS [23, 24]. While a substantial body of literature exists for dextran‐coated SPIONs, reports of highly water‐soluble DS‐coated SPIONs are comparatively fewer. For instance, several dextran‐coated SPIONs such as Feridex and Ferumoxtran‐10 (dextran‐coated), and Resovist (carboxydextran‐coated), were commercially developed for MRI imaging of the reticuloendothelial system and liver [25, 26].

HP‐SPIONs have also been utilized for in vivo tracking of stem cells, whereby mesenchymal stem cells (MSCs) were pre‐treated with HP‐SPIONs in vitro before infusion [23]. A similar strategy was employed to develop thiol‐functionalized HP‐SPIONs for surface labeling of maleimide‐modified pancreatic islets [27]. However, a significant limitation of these conventional coating methods has proven to be the inherent instability and aggregation of SPIONs in saline solutions. To overcome this, studies have demonstrated that polydopamine coatings can enhance SPION stability without inducing toxicity [28]. Additionally, HP‐coated SPIONs have been stabilized by modifying the SPIONs surface with oleic acid, followed by ligand exchange with dopamine‐functionalized HP [21].

**FIGURE 1:**
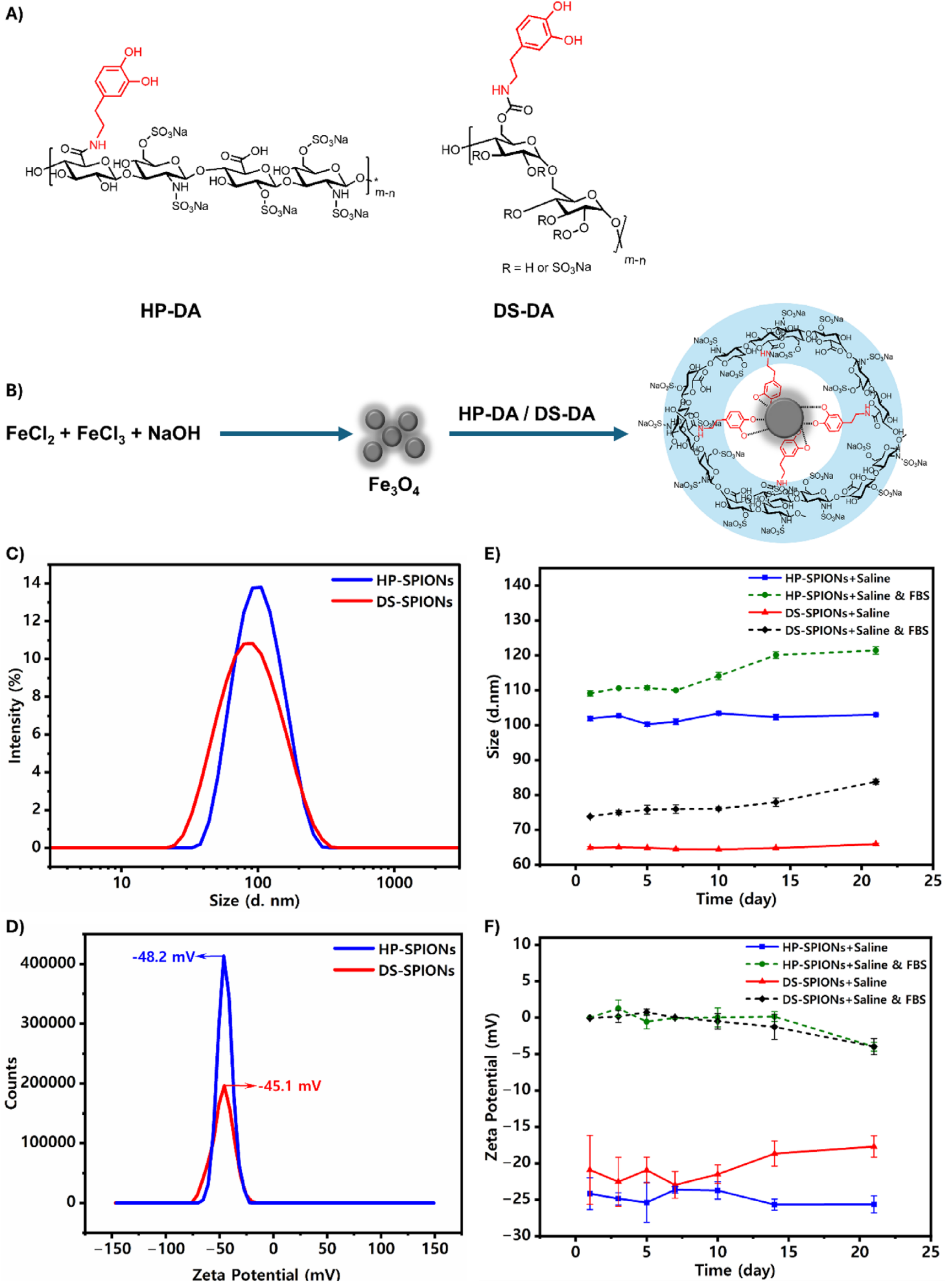
Synthesis of functionalized biopolymer‐coated iron oxide nanoparticles (A) Chemical structure of dopamine functionalized biopolymers, HP‐DA and DS‐DA, (B) Schematic representation of HP‐SPIONs synthesis, (C) hydrodynamic size distribution and (D) zeta potential of DS‐SPIONs and HP‐SPIONs in DIW (1 mg mL^−1^) determined by DLS (E) stability studies as estimated by hydrodynamic size distribution and (F) zeta potential of DS‐SPIONs and HP‐SPIONs in 0.9% saline and 0.9% saline supplemented with 5% FBS over 21 days (1 mg mL^−1^).

Leveraging the unique metal‐binding capabilities of dopamine units (DA) grafted onto DS and HP biopolymers (DS‐DA and HP‐DA) (Figure 1A), we established a robust coating strategy for bare SPIONs. We hypothesized that direct coating of the bare SPIONs surface with DS‐DA and HP‐DA would provide sufficient metal complexation, thereby significantly enhancing their stability in serum and various ionic solutions. The SPIONs were synthesized following the co‐precipitation method in alkaline conditions, which has some advantages over other synthesis methods, including simplicity, good homogeneity, cost‐effectiveness, high product purity, control over particle size and shape [29]. Subsequently, bare synthesized SPIONs were coated with functionalized HP and DS (Figure 1B). For this purpose, dopamine was first grafted to the HP via carbodiimide coupling chemistry by 12.8% conjugation degree with respect to the disaccharide repeat units as estimated by UV–Vis spectroscopy [30]. As DS lacks carboxyl groups, we harnessed the hydroxyl groups present in its backbone, which were activated by carbonyl diimidazole (CDI) to graft dopamine through a stable carbamate linkage. The degree of dopamine modification was estimated to be 5.8% with respect to the sulfated glycosyl units as determined by UV‐Vis spectroscopy (Figure S1A). Additionally, we confirmed the dopamine conjugation to HP and DS by ^1^H NMR spectroscopy recorded in D_2_O and DMSO‐d_6_ at 298 K, respectively. Both DS‐DA and HP‐DA displayed aromatic proton resonances between 6.8–7.2 ppm, consistent with successful covalent grafting to dopamine (Figure S1B,C). The synthesized DS‐DA and HP‐DA were subsequently used to coat freshly synthesized iron oxide nanoparticle cores to form stable biofunctionalized SPIONs with good polydispersity following the co‐precipitation process.

To obtain optimal size with desired stability in buffer, we performed systematic parametric optimization by varying key synthetic conditions, including polymer‐to‐iron core ratio, ultrasonication, and thermal annealing (Table 1 and Table 2). First, we optimized the molar ratio of the polymer to iron oxide core to find the optimal concentration of polyanionic coating for sufficient surface coverage, reflecting improved size distribution and colloidal stability. Specifically, the increase of Fe_3_O_4_:polymer ratio from 1:0.8 to 1:1.6 favorably altered the size of DS‐SPIONs and HP‐SPIONs from 126.7 to 92.6 nm and 134.2 to 118.9 nm, respectively, decreasing visible particle aggregation and lowering polydispersity. However, increasing the proportion to 1:2.5 for DS‐SPIONs was detrimental as it resulted in larger particles but higher surface charge (−59.7 mV) and slightly narrower size distribution, suggesting denser polymeric coverage. To further enhance particle uniformity, probe ultrasonication was introduced. A 30 min sonication step at room temperature moderately decreased the size of DS‐SPIONs to 90 nm and HP‐SPIONs to 128.4 nm by dispersing aggregates and facilitating polymer adsorption. However, extending sonication yielded inconsistent effects attributed to overexposure to shear forces and localized heat at cavitation points, thereby compromising the integrity of the catechol‐Fe complexes and fragmenting the polysaccharide backbone [31]. Consequently, the influence of heating at 90°C was assessed while maintaining a constant polymer ratio (1:1.6) and fixed sonication time (30 min). Refluxing for 30 min produced an optimal monodisperse nanoformulations by balancing between core crystallinity and shell consolidation. The negative surface charge values confirmed dense polyanion grafting and successful suppression of interparticle attractions. Longer thermal exposure tended to increase nanoparticles’ size and reduce their size uniformity, likely due to additional crystal growth and agglomeration, potentially due to mechanisms like Ostwald ripening and oriented attachment. Therefore, under the optimized synthetic conditions, we obtained DS‐SPIONs and HP‐SPIONs with the hydrodynamic size of 78.6 and 92.7 nm, respectively in deionized water, with fairly monodisperse size distribution as measured by DLS (Figure 1C). Moreover, zeta potential analysis indicated highly negative surface charges of −45.1 and −48.2 mV for DS‐SPIONs and HP‐SPIONs, respectively (Figure 1D).

**TABLE 1:** Optimization parameters for the synthesis of stable DS‐SPIONs.

Fe_3_O_4_ [mole]	DS‐DA [mole]^a^	Prob Sonication	Temperature	Size [nm]	PDI	Zeta Potential [mV]
1	0.8	—	RT^b^	126.7	0.387	−28.7
1	2.5	—	RT^b^	108.2	0.269	−59.7
1	1.6	—	RT^b^	92.6	0.326	−45.9
1	1.6	30 min	RT^b^	90.0	0.291	ND^c^
1	1.6	60 min	RT^b^	99.8	0.176	ND^c^
1	**1.6**	**30 min**	**90°C, 30 min**	**78.6**	**0.195**	−**45.1**
1	1.6	30 min	90°C, 60 min	98.9	0.235	ND^c^
1	1.6	30 min	90°C, 90 min	105.5	0.285	ND^c^

^a^ calculated using molecular weight of sulfated glycosyl repeat units,

^b^ Room temperature,

^c^ Not determined.

**TABLE 2:** Optimization parameters for the synthesis of stable HP‐SPIONs.

Fe_3_O_4_ [mole]	HP‐DA [mole]^a^	Prob Sonication	Temperature	Size [nm]	PDI	Zeta Potential [mV]
1	0.8	—	RT^b^	134.2	0.288	−34.0
1	1.6	—	RT^b^	118.9	0.200	−59.0
1	1.6	30 min	RT^b^	128.4	0.230	ND^c^
1	1.6	60 min	RT^b^	146.3	0.242	ND^c^
1	**1.6**	**30 min**	**90°C, 30 min**	**92.7**	**0.146**	−**48.2**
1	1.6	30 min	90°C, 60 min	137.9	0.172	ND^c^
1	1.6	30 min	90°C, 90 min	179.4	0.159	ND^c^

^a^ calculated using molecular weight of disaccharide repeat,

^b^ Room temperature,

^c^ Not determined.

The DS‐SPIONs and HP‐SPIONs displayed excellent colloidal stability in 0.9% saline and 0.9% saline supplemented with 5% fetal bovine serum (FBS) without any aggregation over 21‐day attributed to the stable polyanionic coatings (Figure 1E,F). Both formulations revealed monodispersed characteristics with stable hydrodynamic size throughout this duration, approximately ∼64 nm for DS‐SPIONs and ∼102 nm for HP‐SPIONs. The surface charge or the zeta potential also reduced from −45.1 mV to −20 mV for DS‐SPIONs and −48.2 to −24 mV for HP‐SPIONs when measured in saline. The smaller size and surface charge in saline compared to deionized water (DIW) is associated with electrostatic assembly and compaction at higher ionic strength, leading to a reduction in the thickness of the hydration layer and the surface charge [32]. Incubation of SPIONs in saline with serum increased the size from ∼64 to ∼75 nm for DS‐SPIONs and ∼102 to ∼110 nm for HP‐SPIONs respectively, presumably due to the adsorption of serum proteins forming a dynamic protein corona. No signs of aggregation were observed over the period of 21 days. Formation of protein corona was further confirmed by the zeta potential measurements as the surface charge of both DS‐SPIONs and HP‐SPIONs in saline with 5% serum. Upon incubation with serum, the initially negative surface charge of the nanoparticles was rapidly neutralised, with zeta potentials shifting from −20 mV (DS‐SPIONs) and −24 mV (HP‐SPIONs) to approximately 0 mV immediately after exposure. Over time, the surface charge further decreased, reaching ~−4 mV by day 21. Notably, these findings confirm that dopamine‐grafted HP and DS provide robust steric‐electrostatic stabilization, effectively preventing aggregation even under physiologically complex environments.

**FIGURE 2:**
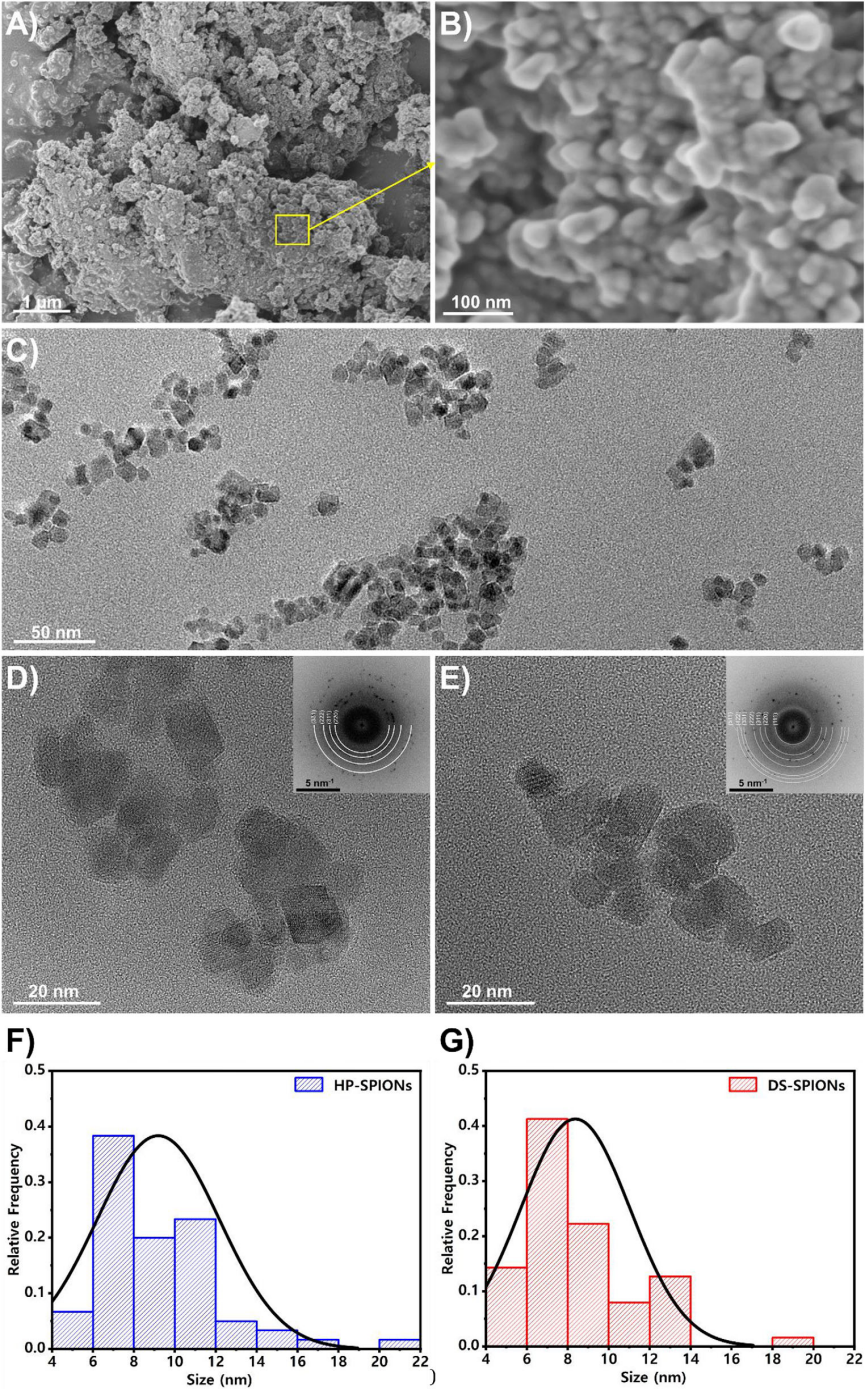
Electron microscopy analysis of SPIONs. (A) Low‐magnification and (B) high‐magnification FESEM images of bare SPIONs. (C) Representative low‐magnification TEM image of surface‐coated SPIONs. (D,E) High‐resolution TEM images of HP‐ and DS‐SPIONs, respectively. The inset images show the corresponding FFT patterns. (F,G) The average sizes of nanoparticles’ core for HP‐ and DS‐SPIONs are 9.2 ± 3 and 8.4 ± 2.7 nm (Average ± SD).

The morphology and dispersion of the synthesized iron oxide nanoparticles were investigated using electron microscopy. Figure 2A,B display field emission scanning electron microscopy (FESEM) images of the pristine, uncoated nanoparticles. At low magnification (Figure 2A), the nanoparticles are observed to form extensive, dense agglomerates. This is attributed to strong interparticle magnetic and van der Waals forces inherent to unmodified nanoparticles [33]. The higher magnification image (Figure 2B) further reveals the nature of these agglomerates, where individual particles are tightly bound, potentially indicating the onset of coalescence. The primary nanoparticles exhibit a blocky and quasi‐spherical morphology.

In contrast, the effect of surface modification is evident in the transmission electron microscopy (TEM) images. Figure 2C shows a representative low‐magnification TEM image of the coated SPIONs, which exhibit a significantly improved state of dispersion on the TEM grid compared to their uncoated counterparts. The surface coating appears to provide effective stabilization, mitigating the tendency for agglomeration in consistent with the studies of coated‐SPIONs [33, 34]. However, some degree of aggregation is typical of sample preparation by evaporation‐induced capillary forces while drying suspension droplet on grid and magnetic dipoles interactions that attract SPIONs together [35, 36, 37].

The moderate agglomeration in TEM images is due to high magnification TEM images of SPIONs provided further insight into the nature of the coated nanoparticles. Figure 2D,E present TEM images of the HP‐coated and DS‐coated SPIONs, respectively. The inset Fast Fourier Transformation (FTT) patterns of the core of coated SPIONs indicate the crystalline lattice planes of the nanoparticles match the distinct diffraction patterns of magnetite with cubic crystalline structure (Fe_3_O_4_‐(PDF5 No. 04‐015‐8203), confirming the successful synthesis of the target crystalline phase. However, direct visualization of the coating layer using conventional TEM imaging was challenging [38], likely due to the high‐energy beam sensitivity of the polymer. These organic biopolymers are susceptible to beam‐induced damage [39], which complicates accurate assessment of coating thickness and conformational structure on the nanoparticle surface. Image analysis of several TEM images was performed to determine the particle size distribution. The apparent average sizes of nanoparticles for HP‐ and DS‐SPIONs were close, measured as 9.2 ± 3 and 8.4 ± 2.7 nm by image analysis, respectively (Figure 2F,G).

Next, we performed thermogravimetric analysis (TGA) to evaluate the polymer content and binding characteristics. The bare SPIONs revealed a weight loss of 3.24% between temperatures ranging from 25–900°C, implying the presence of surface‐adsorbed water molecules on the nanoparticles. DS‐SPIONs and HP‐SPIONs both showed an initial ∼10% weight loss between 40–200°C due to dehydration, followed by significant degradation between 200–650°C that corresponded to oxidation of the polysaccharide backbone and functional groups. The final residual mass at 900°C, representing iron content, was approximately 53% for DS‐SPIONs and 33% for HP‐SPIONs (Figure S1D). These findings confirm the successful fabrication of stable polyanion‐coated SPIONs with structurally comparable and tunable bio‐interface properties, providing functional nanoplatform to investigate the differential biological and immunological responses of HP and DS.

### 2.2 Evaluation of Biocompatibility and Innate Immune Activation of HP‐ and DS‐Coated SPIONs in Human Whole Blood with Endothelial Cells

**FIGURE 3:**
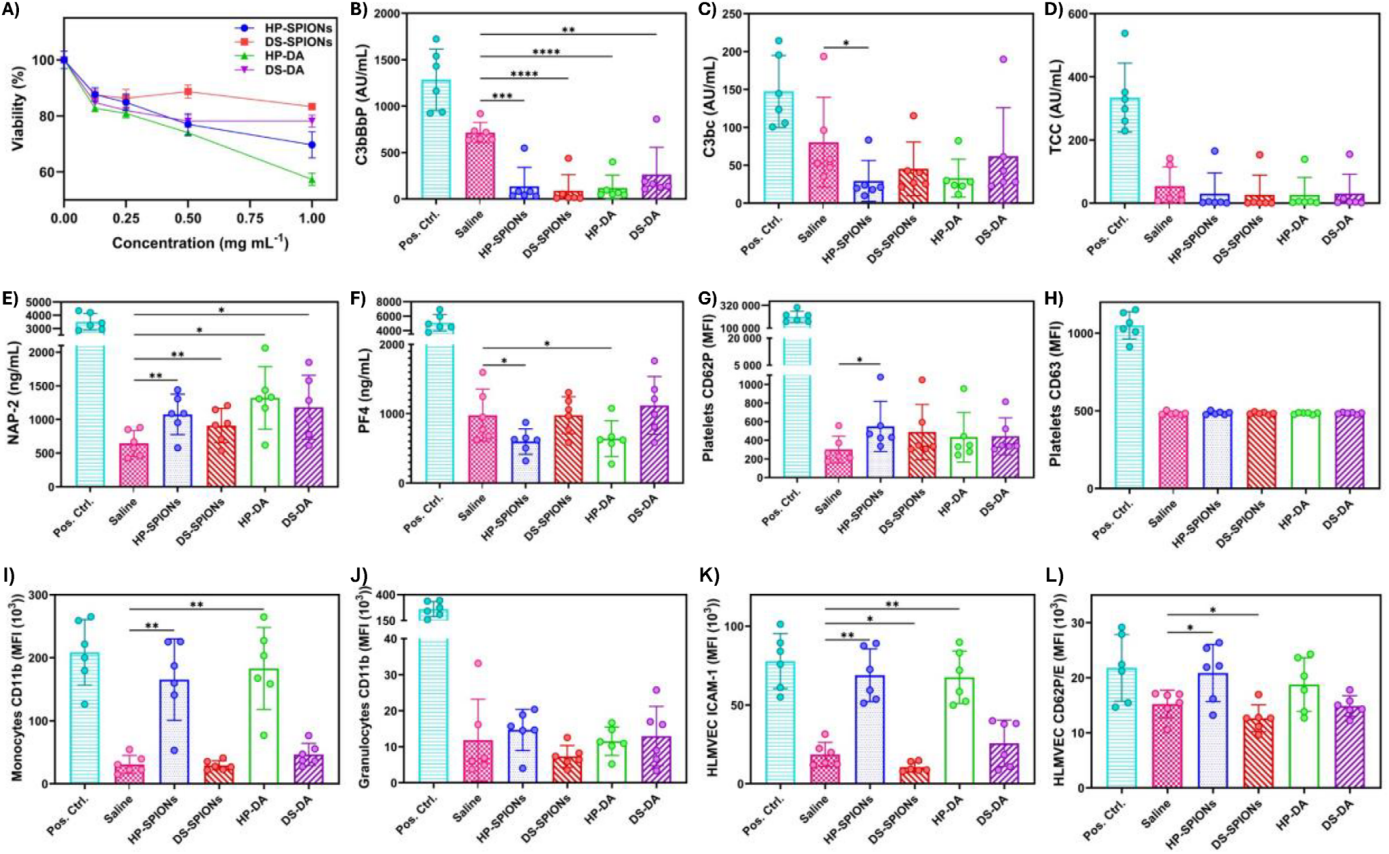
Biocompatibility Evaluation of nanoparticles. (A) Viability assay of HP‐ and DS‐SPIONs, or DS‐DA and HP‐DA on mouse embryonic fibroblast cells were incubated with at 0–1 mg mL^−1^ concentration for 48 h using Alamar Blue (Data represent mean ±SD of *n* = 3). Effects of SPIONs on the complement system, platelets, monocytes, granulocytes and endothelial cells. Human lepirudin‐anticoagulated whole blood was incubated with HP‐DA, DS‐DA, or SPIONs coated with HP or DS, all at 200 µg ml^−1^. A mix of 100 µg ml^−1^ zymosan, 10 ng ml^−1^ LPS, and 25 µg ml^−1^ TRAP‐6 was used as a positive control and saline as a buffer control. (B) Complement (C) C3bBbP, (D) C3b, and (F) TCC and platelet markers (E) NAP‐2 and (F) PF4 were measured in plasma using ELISA, after the full 4 h of incubation. (G) Platelet activation marker CD62P and (H) CD63 expression were measured after 1 h of incubation by flow cytometry. (I) Monocyte and (J) granulocyte CD11b activation were measured using flow cytometry after 15 min of incubation. (K) HLMVEC ICAM‐1 and (L) CD62P/E activation were measured using flow cytometry after 4 h of incubation. Statistical analysis was performed using one‐way ANOVA with Dunnett's multiple‐comparison post‐hoc test (*n* = 6, *p*<0.05 (*), *p*<0.01 (**), *p*<0.001 (***), *p*<0.0001 (****)).

We first performed the cytotoxicity of DS‐SPIONs and HP‐SPIONs using mouse embryonic fibroblast cell lines via the Alamar Blue assay following manufacturer's protocol. The DS‐SPIONs and HP‐SPIONs were suspended in cell culture medium (0–1 mg mL^−1^ concentration) and dose escalation study was performed by serial dilution. We observed cell viability of ∼80%–90% when DS‐SPIONs and HP‐SPIONs were incubated with cells for 48 h (up to 0.5 mg mL^−1^ concentration) (Figure 3A). In subsequent in vitro studies, we used 200 µg mL^−1^ concentration as this dose was not detrimental to cell viability. Moreover, 200 µg mL^−1^ dose represents the anticipated intravascular level of SPIONs following an IV dose of 5 mg [Fe] kg^−1^ that is commonly used in drug delivery and MRI applications [37, 40]. Using standard IV‐bolus calculations (C_0_ = Dose/V_d_; mouse blood volume 60–80 mL kg^−1^), this dose corresponds to an estimated initial iron concentration of ∼62–83 µg [Fe] mL^−1^ [3]. Converting this to nanoparticle mass using our TGA‐determined iron fractions (*f_Fe_* = 0.24 for HP‐SPIONs and 0.38 for DS‐SPIONs) yields approximately 0.2 mg mL^−1^, thereby justifying the use of 200 µg mL^−1^ as a physiologically relevant in vitro exposure level.

Then, we evaluated their hemocompatibility and thromboinflammatory potential to predict the in vivo behavior and assess the immediate biological impact of our nanoparticles. For this purpose, we utilized a clinically relevant ex vivo model where the nanoparticles were incubated in lepirudin‐anticoagulated human whole blood together with human lung microvascular endothelial cells (HLMVECs). This section details the systematic analysis of key innate immune responses within this model.

The clinical utility of nanocarriers is significantly hampered by their rapid sequestration through the reticuloendothelial system (RES). This clearance is primarily initiated by complement activation, which opsonizes the nanoparticles (C3b/iC3b) for phagocytic uptake and generates C5a anaphylatoxin, a potent driver of thromboinflammation. This innate immune cascade involves a complex crosstalk between monocytes, neutrophils, and platelets that promotes thrombosis and inflammation [41]. Activated platelets exacerbate this by releasing chemokines like platelet factor 4 (PF4, CXCL4) and neutrophil‐activating peptide‐2 (NAP‐2, CXCL7), which activate neutrophils and endothelial adhesion [42]. Consequently, evaluating the relative ‘stealth’ properties of DS and HP in evading these innate immune pathways is imperative for identifying biocompatible drug delivery systems.

To perform this experiment, whole blood was collected from six healthy volunteers and the activation of complement, platelets, monocytes, granulocytes and HLMVECs were analyzed using ELISA and flow cytometry. We determined the level of complement activation after incubation with DS‐SPIONs and HP‐SPIONs for 4 h, as analyzed by expression of complement activation markers C3bc, C3bBbP, and the soluble C5b‐9 terminal complement complex (TCC). The polymer precursors, namely dopamine‐conjugated HP (HP‐DA) and DS (DS‐DA) were used as a control to understand the difference in immune response as a result of nanoformulation. We discovered that both HP‐SPIONs and DS‐SPIONs suppressed complement activation to a similar extent. Both SPIONs and the biopolymers significantly reduced C3bBbP (Figure 3B), indicating specific regulation of complement at the alternative pathway convertase, presumably by recruiting factor H. Similarly, C3‐cleavage products C3bc (Figure 3C) and TCC (Figure 3D) were significantly reduced relative to saline control and positive control (mixture of zymosan, LPS, and TRAP‐6). These observations are consistent with the capacity of highly sulfated polysaccharides to recruit the soluble regulator factor H, thereby accelerating decay of the alternative pathway C3 convertase and acting as ‘dysopsonins’ for nanoparticles [43, 44].

Our results clearly demonstrated that both HP and DS possess excellent ability to attenuate complement system activation and that the nanoformulation did not induce any detrimental effects on their bioactivity. This reduction in complement activation is crucial, as it not only prevents the release of C3a/C5a anaphylatoxins, limiting acute inflammation, but also avoids rapid C3b‐mediated clearance of nanoparticles by phagocytes [45]. The surface modification of SPIONs with sulfated biopolymers therefore provides a stealthier corona that resists complement opsonization, in contrast to traditional dextran‐coated iron oxide nanoparticles which readily triggers the alternative pathway [45].

Next, we estimated the extent of platelet activation by DS‐SPIONs and HP‐SPIONs in human whole blood. We observed that the levels of expression of platelet α‐granule NAP‐2 (Figure 3E) were slightly elevated (*p*<0.05‐*p*<0.01) upon incubation with DS‐SPIONs and HP‐SPIONs as well as their dopamine conjugated precursors, indicating some platelet activation. Interestingly, the α‐granule PF4, a chemokine that typically follow the pattern of NAP‐2 [46], was decreased (*p*<0.05) upon incubation with HP‐DA and HP‐SPIONs relative to DS‐SPIONs (Figure 3F). This might be more an effect of polyanions, and especially heparin, forming complexes with PF4, which could cause epitope masking [47]. HP‐SPIONs slightly increased (*p*<0.05) the MFI of the α‐granule CD62P (P‐selectin), but no effect was evident for the dense granule CD63 for any of the conditions (Figure 3G,H). Taken together, the platelet marker analysis indicated a minor α‐granule, but no dense granule release.

HP‐SPIONs and HP increased monocyte CD11b expression significantly (*p*<0.01), whereas DS and DS‐SPIONs stimulated no increase in monocyte CD11b activation (Figure 3I,J). HLMVEC activation was measured through ICAM‐1 and CD62P/E expressions. ICAM‐1 MFI increased (*p*<0.01) when HP and HP‐SPIONs were added, whereas activation was decreased (*p*<0.05) by DS‐SPIONs treatment (Figure 3K). CD62P/E followed a less strong, but similar pattern, where HP‐SPIONs increased (*p*<0.05) and DS‐SPION decreased (*p*<0.05) the CD62P/E MFI (Figure 3L).

The elevated expression of CD11b and ICAM‐1 induced by HP and HP‐SPIONs treatment, despite attenuated complement activation, suggests a complement‐independent mechanism likely mediated through direct interactions of heparin with innate immune receptors such as TLR4, which are known to rapidly promote leukocyte and endothelial cell activation via intracellular signaling pathways [48, 49]. This can suppress inflammation and immune responses. Conversely, DS‐SPIONs reduced endothelial ICAM‐1 and E‐selectin expression, consistent with reports that DS functions as an endothelial protectant, a human complement inhibitor, and a suppressor of natural killer cell‐mediated cytotoxicity against porcine cells [50].

Overall, surface modification of SPIONs with highly sulfated polysaccharides modulates key immune and endothelial responses, enabling reduced complement activation and controlled platelet cellular activation in human blood. These findings underscore the importance of surface engineering to enhance nanoparticles biocompatibility and to minimize the pro‐inflammatory and thromboinflammatory risks in biomedical applications.

### 2.3 Immunological Responses to SPIONs in Murine Immune Cells

**FIGURE 4:**
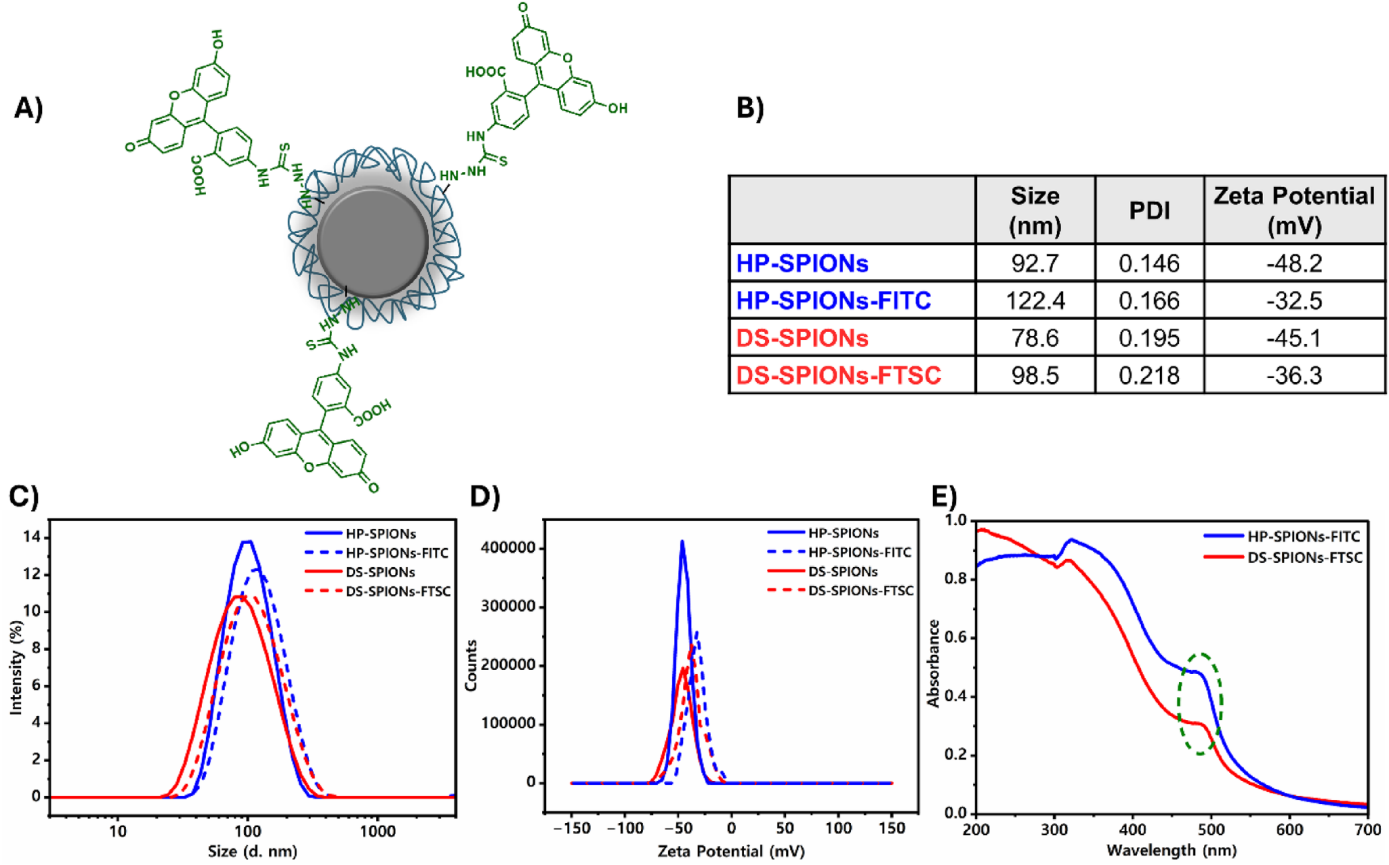
Fluorescent labeling of nanoparticles. (A) Schematic chemical structure of fluorescently labeled DS‐SPIONs. (B) Table summarizing hydrodynamic size and zeta potential of fluorescent and non‐fluorescent nanoparticles. (C) Hydrodynamic size distribution and (D) zeta potential of fluorescent‐labeled SPIONs and coated SPIONs in DIW (1 mg mL^−1^) determined by DLS (E) UV–vis spectra of fluorescent SPIONs dissolved in DIW (1 mg mL^−1^).

We next investigated the relative preference of DS‐SPIONs and HP‐SPIONs to target specific immune cells by quantifying the uptake of fluorescently labeled SPIONs in primary murine immune cells. To perform this study, fluorescently labeled DS‐SPIONs and HP‐SPIONs were synthesized. Specifically, HP‐SPIONs‐FITC were synthesized by covalently grafting fluorescein isothiocyanate (FITC) to the hydrazide functionalized HP‐SPIONs decorated on the nanoparticle surface that form a stable thiourea linkage following our previously optimized protocol [51]. To design fluorescently labeled DS‐SPIONs, we followed a Micheal addition strategy, whereby we first incorporated methacrylate groups on the DS‐SPIONs as a molecular handle to conjugate fluorescein thiosemicarbazide (FTSC) (Figure 4A). As a control group we directly conjugated an FTSC group to HP (HP‐FTSC) and DS polymers (DS‐FTSC) to understand the impact of nanoformulations on immune uptake.

Conjugation of fluorescein molecules on DS‐SPIONs and HP‐SPIONs increased their hydrodynamic size from 78.6 to 98.5 nm, and 92.7 to 122.4 nm, respectively (Figure 4B,C). Fluorescein conjugation also reduced the surface charge on the nanoparticle surface as the zeta potential reduced from −45.1 to −36.3 for DS‐SPIONs‐FTSC, and from −48.2 to −32.5 for HP‐SPIONs‐FITC (Figure 4B,D). The degree of fluorescent conjugation in HP‐SPIONs and DS‐SPIONs was estimated to be 9.6% and 5.9% by UV–Vis spectroscopy with respect to molecular weight of HP and DS (15 kDa) using the FITC and FTSC extinction coefficient of 75 000 and 78 000 m^−1^ cm^−1^, respectively (Figure 4E). In addition, the fluorescent measurement confirmed the labeling of FITC on HP‐SPIONs at 492 nm wavelength and FTSC on DS‐SPIONs at 495 nm wavelength (Figure S1E).

**FIGURE 5:**
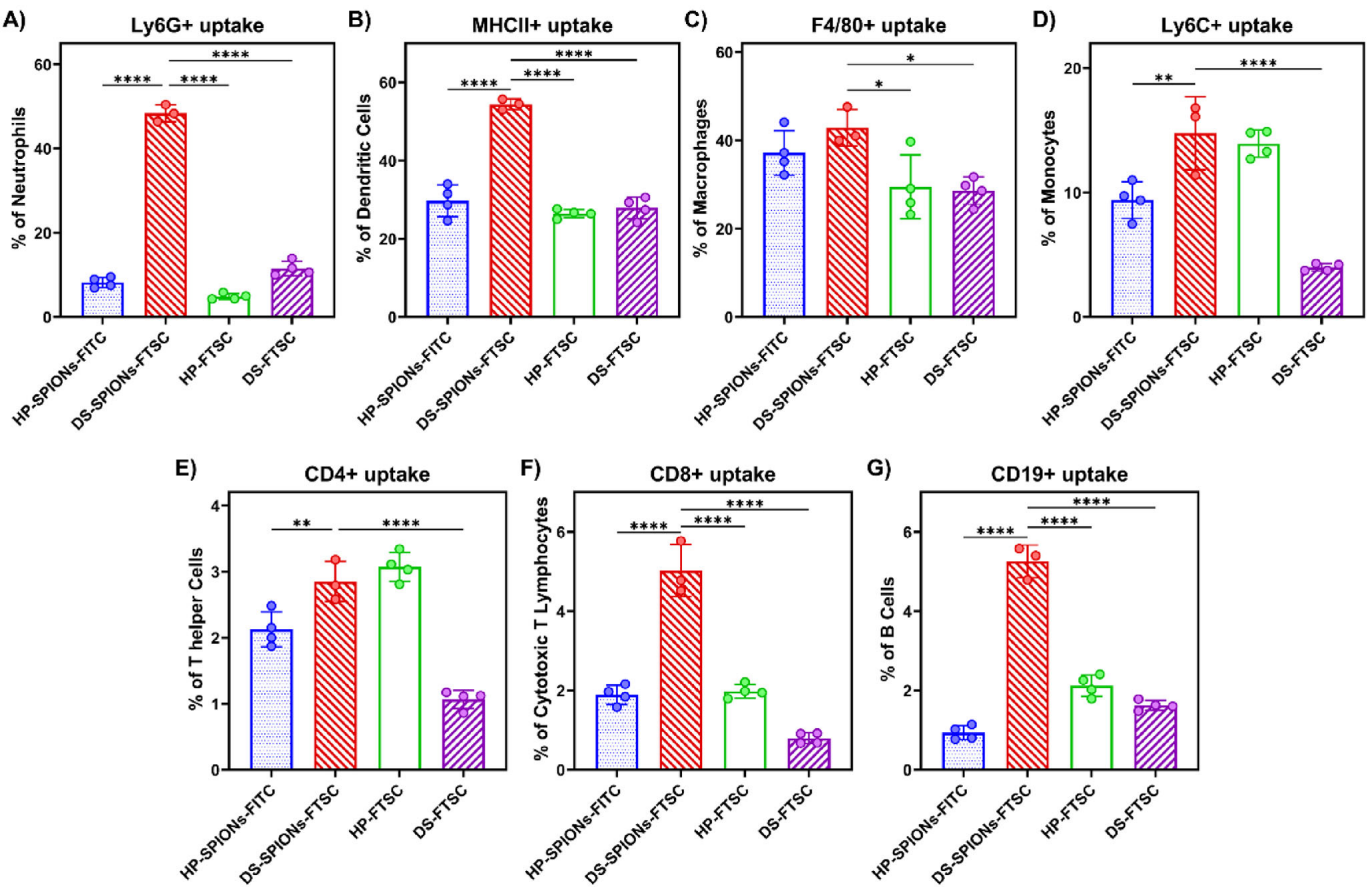
Immune cell uptake of DS‐ and HP‐coated SPIONs by splenic immune cell subsets. Murine spleen‐derived immune cells were incubated *ex vivo* with fluorescently labeled SPIONs (DS‐SPIONs and HP‐SPIONs), and polymers (DS and HP) for 4 h at 37°C and uptake was assessed by flow cytometry. Data are presented as the proportion of (A) neutrophils (Ly6G⁺), (B) dendritic cells (MHC II⁺), (C) macrophages (F4/80⁺), (D) monocytes (Ly6C⁺), (E) CD4⁺ T cells, (F) CD8⁺ T cells, and (G) B cells (CD19⁺) that have engulfed the nanoparticles. DS‐SPIONs had a significantly higher uptake in phagocytic myeloid populations compared to polymers alone or HP‐SPIONs, respectively, with minimal uptake observed in lymphocyte subsets. Data represent mean ±SD of *n* = 3–‐4 individual animals per group. Statistical analysis was performed by one‐way ANOVA with Tukey's post‐hoc test (*p*<0.05 (*), *p*<0.01 (**), *p*<0.001 (***), *p*<0.0001 (****)).

Following successful fluorescein labeling, we incubated the nanoparticles with murine splenic cell extracts for 4 h and evaluated their uptake by various immune cell populations using flow cytometry. Since splenic cell suspensions contain a diverse array of immune cells, including granulocytes, monocytes, macrophages, dendritic cells (DCs), natural killer (NK) cells, T cells, and B cells, this approach enabled us to assess the preferential uptake of nanoparticles across different cell types. Our results revealed that DS‐SPIONs‐FTSC were taken up more efficiently by myeloid cells than HP‐SPIONs‐FITC. This is evident from the fact that DS‐SPIONs‐FTSC displayed ∼50% uptake by Ly6G neutrophils, ∼55% of MHC II⁺ DCs, and ∼42% of F4/80⁺ macrophages (Figure 5A–C). In contrast, HP‐SPIONs were taken up by <25% of DCs and only 5% of neutrophils. The higher uptake by DS‐SPIONs could be attributed to its high‐affinity for scavenger receptor class A (SR‐A, MARCO) and complement receptors CR3 (CD11b/CD18) that are expressed on myeloid cells. The uptake patterns suggest that the DS coating on DS‐SPIONs facilitates binding to phagocytic receptors in splenic innate cells, while the HP corona on HP‐SPIONs minimizes immune recognition and uptake. Thus, HP‐SPIONs exhibited minimal engagement with myeloid populations and effectiveness on lower visibility to immune surveillance under non‐inflammatory conditions, providing them with potential stealth properties.

Monocytes (Ly6C^+^) also exhibited an increased uptake of DS‐SPIONs compared to HP‐SPIONs and DS‐FTSC, while the uptake of HP‐FTSC was comparable to that of DS‐SPIONs (Figure 5D). Unlike myeloid cells, uptake by lymphoid cells was limited. Nevertheless, a small proportion of T and B cells showed uptake of DS and HP particles, with a relatively higher proportion of DS‐SPIONs being taken up by CD4⁺, CD8⁺ T cells, and CD19⁺ B cells (Figure 5E–G). Our in vitro uptake study indicates a superior uptake of DS‐SPIONs by both myeloid and lymphoid cells, revealing its potential for targeted delivery to immune cells.

### 2.4 Immunomodulatory Response of SPIONs in Primary Murine Macrophages

The immune cell uptake levels revealed macrophages as a key population interacting with both SPIONs formulations. Given the significant difference in internalization efficiency between DS‐SPIONs and HP‐SPIONs, we next evaluated whether these biopolymer‐coated SPIONs differed in their macrophage‐polarizing capabilities at the transcriptional level. Primary bone marrow‐derived macrophages (BMDMs) were isolated from healthy C57BL/6 mice and cultured according to established protocols [52]. Cells were treated with DS‐SPIONs, HP‐SPIONs, and their respective dopamine‐functionalized precursors, DS‐DA and HP‐DA.

**FIGURE 6:**
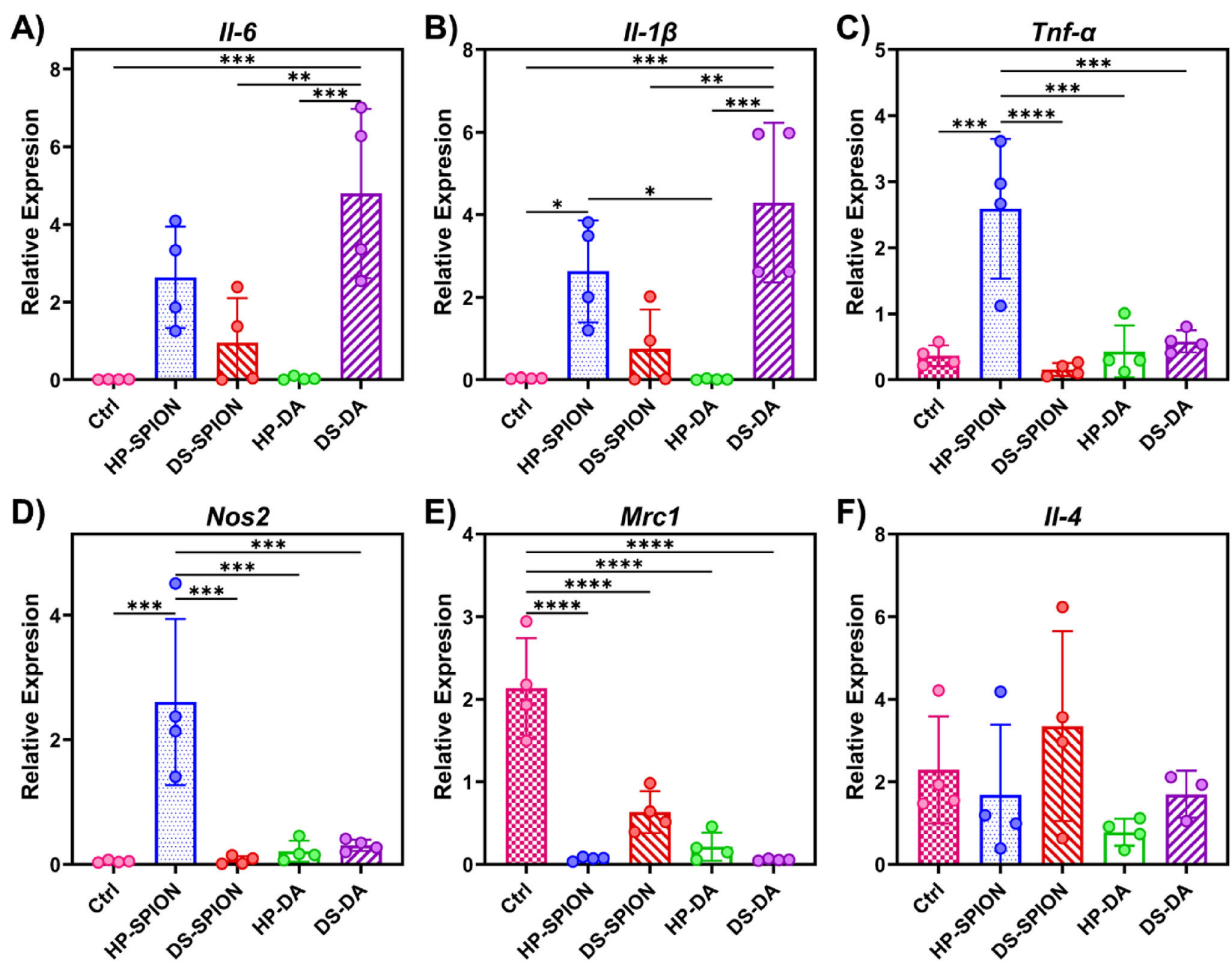
Macrophage polarization of murine macrophages in response to biopolymer‐coated SPIONs and their dopamine‐functionalized precursors. Relative expression of (A) *Il‐6*, (B) *Il‐1β*, (C) *Tnf‐α*, (D) *Nos2*, (E) *Mrc1*, (F) *Il‐4*, in primary BMDMs treated with DS‐SPIONs, HP‐SPIONs, DS‐DA, and HP‐DA for 24 h. Gene expression was measured by RT‐qPCR and normalized to *Hprt*. Data are presented as mean ± SD. Statistical analysis was performed using one‐way ANOVA with Tukey's post‐hoc test (*p*<0.05 (*), *p*<0.01 (**), *p*<0.001 (***), *p*<0.0001 (****)).

Interestingly, DS‐DA induced a pronounced pro‐inflammatory response characterized by elevated *Il‐6* and *Il‐1β* mRNA expression (Figure 6A,B). However, DS‐SPIONs suppressed this response, as evidenced by reduced *Tnf‐α* and *Nos2* levels, along with modest increased expression of *Mrc1* and *Il‐4*, markers associated with an anti‐inflammatory phenotype (Figure 6C–F). In contrast, HP‐SPIONs significantly enhanced the pro‐inflammatory M1‐like responses compared to HP‐DA by upregulation of *Nos2*, *Il‐1β*, *Tnf‐α*, and *Il‐6* expressions, aligning with increased endothelial cells activation characterized in whole blood assays (Figure 6A–D). Elevated *Il‐1β* and *Tnf‐α* synergistically induce adhesion molecules such as ICAM‐1 and E‐selectin on endothelial cells, facilitating monocyte recruitment in inflammatory settings [53].

The divergent immunological profiles of HP‐ and DS‐SPIONs likely stem from their distinct receptor interactions and the multivalent nature of the nanoparticles surface. While HP is widely used for its anticoagulant properties, surface‐immobilized on HP‐SPIONs creates a high‐density, multivalent interface. This presentation facilitates the clustering of innate immune receptors, such as Mac‐1 (CD11b/CD18) and TLR4, which are known to trigger pro‐inflammatory signaling cascades (e.g., NF‐κB) upon cross‐linking [48, 54]. This aligns with the observed upregulation of pro‐inflammatory cytokines (*Tnf‐α*, *Il‐1β*) in our study. Conversely, DS is a well‐characterized ligand for SR‐A, facilitating the rapid silent clearance of ligands without eliciting an inflammatory response [12, 55]. The high uptake of DS‐SPIONs by myeloid cells, coupled with the suppression of inflammatory markers, suggesting that DS‐SPIONs engage these non‐phlogistic clearance pathways.

**FIGURE 7:**
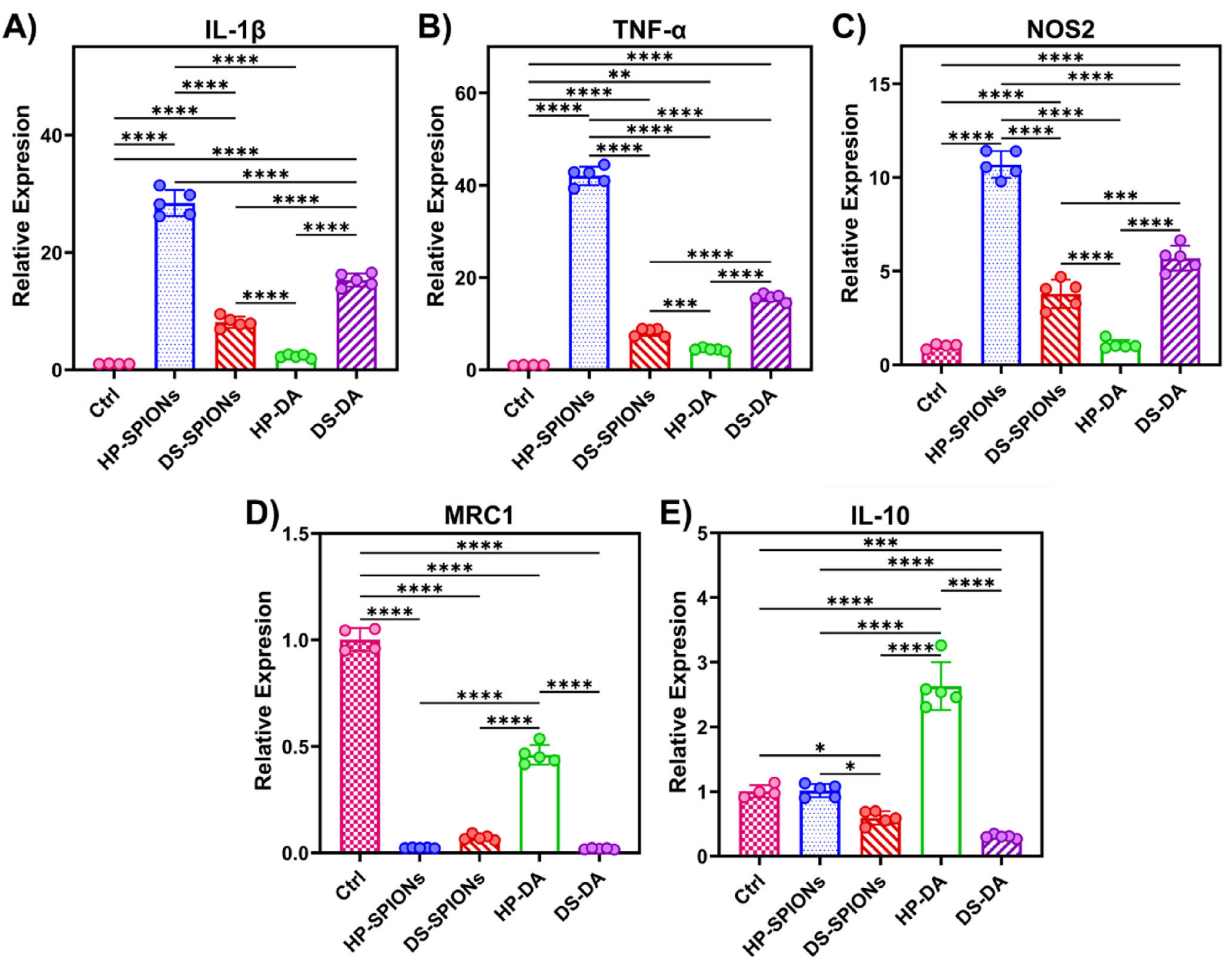
Macrophage polarization of human macrophages in response to biopolymer‐coated SPIONs and their dopamine‐functionalized precursors. Relative expressions of (A) IL‐1β, (B) TNF‐α, (C) NOS2, (D) MRC1, (E) IL‐10, in M0 differentiated from THP‐1 treated with DS‐SPIONs, HP‐SPIONs, DS‐DA, and HP‐DA for 24 h. Gene expression was measured by RT‐qPCR and normalized to GAPDH. Data are presented as mean ± SD. Statistical analysis was performed using one‐way ANOVA with Tukey's post‐hoc test (*p*<0.05 (*), *p*<0.01 (**), *p*<0.001 (***), *p*<0.0001 (****)).

The macrophage polarization response was further examined in naïve human macrophages (M0) differentiated from THP‐1 monocytic cells. Treatment with HP‐SPIONs and DS‐DA demonstrated a pronounced upregulation of pro‐inflammatory markers (IL‐1β, TNF‐α, and NOS2) and a concurrent profound downregulation of MRC1, indicative of a robust M1‐like phenotype that reflects the polarization profile observed in primary BMDMs (Figure 7A–D). Subsequently, we differentiated naïve M0 macrophages to pro‐inflammatory M1 using lipopolysaccharide (LPS) and interferon gamma (IFN‐γ) and anti‐inflammatory M2 macrophages using Interleukin‐4 (IL‐4) and estimated the cytokine expression levels after incubation with polymer coated SPIONs. As anticipated, incubation of polymer coated SPIONs with pre‐polarized macrophages displayed elevated IL‐1β and TNF‐α expression, especially by HP‐SPIONs in both M1 and M2 polarized macrophages, reinforcing the moderate pro‐inflammatory potential of the nanomaterials (Figure S2A,B,D,E). Fascinatingly, HP‐DA treatment significantly increased IL‐10 expression while maintaining IL‐1β, TNF‐α, and NOS2 at baseline, reflecting an anti‐inflammatory M2‐like phenotype (Figure 7E; Figure S2C,F). The immunological response of HP‐NPs is complex and fascinating as exposure of immune cells to Low molecular weight heparin (LMWH) is reported to increase macrophage expression of HLA‐DR and CD206 and elevates secretion of the Th17‐associated chemokine CCL20, indicating that LMWH drives macrophage activation toward a pro‐inflammatory phenotype [56]. However, HP coated biomaterial surfaces [57] and HP‐coated SPIONs loaded with bFGF2 [58] elicit M2‐like macrophage polarization or display anti‐inflammatory effects. Thus, the biopolymer assembly and presentation to the immune cells strongly influence the cytokine responses, demonstrating a clear difference between linear HP‐DA and HP‐SPIONs that is potentially mediated by the difference in cellular uptake and subsequent activation.

**FIGURE 8:**
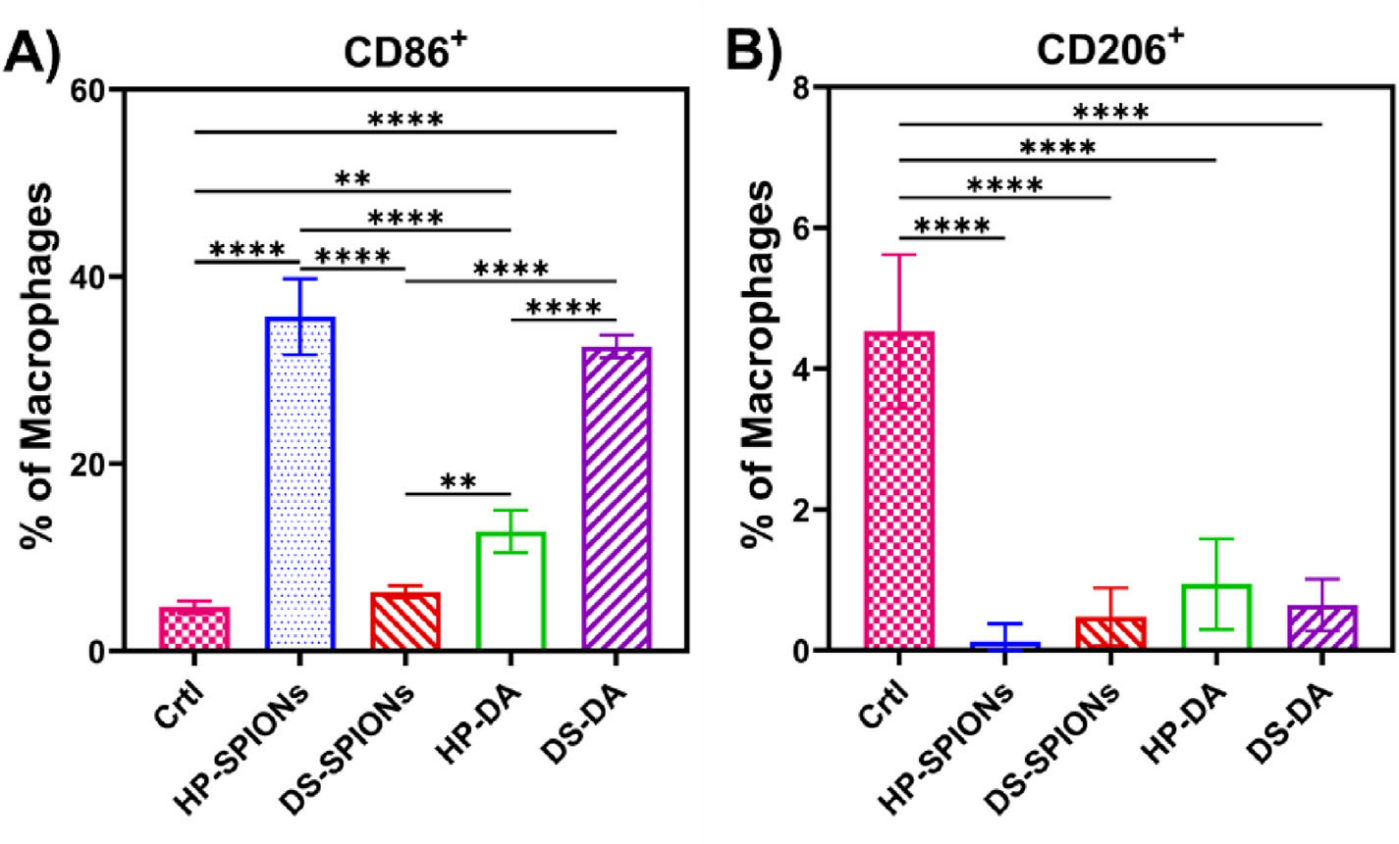
Protein level expression of murine macrophages in response to biopolymer‐coated SPIONs and their dopamine‐functionalized precursors. Relative expression of (A) CD86^+^, (B) CD206^+^. Protein level was measured by flow cytometry. Data are presented as mean ± SD. Statistical analysis was performed using one‐way ANOVA with Tukey's post‐hoc test (*p*<0.05 (*), *p*<0.01 (**), *p*<0.001 (***), *p*<0.0001 (****)).

To further assess macrophage polarization toward M1‐like or M2‐like phenotypes, we performed flow cytometry following a 24 h incubation of murine BMDMs with DS‐SPIONs, HP‐SPIONs, or their dopamine‐functionalized precursors (DS‐DA and HP‐DA). CD86 and CD206 were selected as hallmark markers of M1 and M2 activation, respectively. Consistent with the elevated *Nos2*, *Il‐1β*, *Tnf‐α*, and *Il‐6* mRNA expression, HP‐SPIONs and DS‐DA markedly increased CD86 levels, confirming a pro‐inflammatory M1‐like activation (Figure 8A). In contrast, no upregulation of CD206 was observed at 24 h (Figure 8B). The limited detection of M2‐associated markers may be attributed to enzymatic cleavage of cell‐surface proteins during the cell detachment process [59].

Collectively, these results indicate that, unlike DS‐SPIONs, HP‐SPIONs exhibit a more pronounced pro‐inflammatory profile, in agreement with our qRT‐PCR findings. Specifically, HP‐SPIONs and DS‐DA drive M1‐like polarization, whereas DS‐SPIONs and HP‐DA maintain macrophages in a transitional state without inducing classical M2 polarization.

### 2.5 Immune Response of SPIONs in Healthy Mice

**FIGURE 9:**
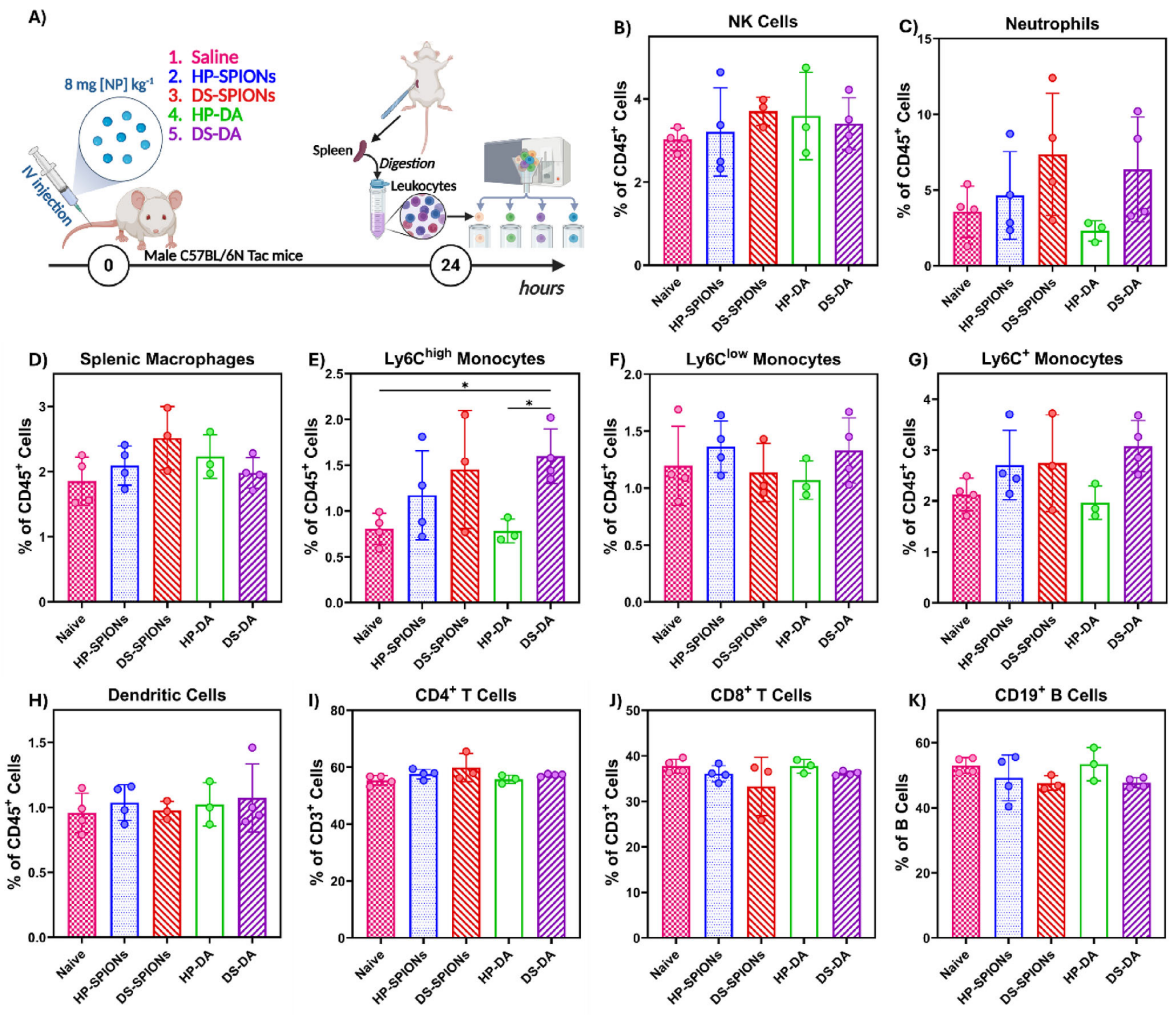
Influence of bioconjugated nanoparticles on mice blood cells. (A) Schematic representation of experimental design. Healthy mice were intravenously injected with 8 mg kg^−1^ of DS‐SPIONs and HP‐SPIONs, and their dopamine‐functionalized precursors(DS‐DA, HP‐DA). Spleens were collected 24 h post‐injection, processed into single‐cell suspensions, and analyzed via multiparametric flow cytometry. Quantification of immune cells in the spleen: (B) NK cells, (C) neutrophils (Ly6G^+^), (D) splenic macrophages (F4/80^+^), (E) Ly6C^+high^ monocytes, (F) Ly6C^+low^ monocytes, (G) Ly6C^+^ monocytes, (H) dendritic cells (CD11c^+^), (I) CD4^+^ T cells, (J) CD8^+^ T cells, (K) CD19^+^ B cells. Data are presented as percentage of CD45^+^ cells, representing mean ± SD using individual animals (*n* = 3–4 per group). Statistical analysis was performed using one‐way ANOVA with Dunnett's multiple comparisons test (*p*<0.05 (*)).

To further elucidate whether DS‐ or HP‐coated SPIONs affect the innate and adaptive immune cell populations after infusion in vivo, we isolated the immune cells from mouse spleens 24 h post intravenous administration and analyzed the cell populations by flow cytometry (Figure 9A). Our flow cytometric analysis highlighted distinct immunological responses associated with the nanoparticle coatings. DS‐SPIONs induced a modest, yet statistically non‐significant, increase in the frequencies of NK cells (1.22‐fold, *p*‐value 0.604), Ly6G⁺ neutrophils (2.06‐fold, *p*‐value 0.251), F4/80⁺ macrophages (1.36‐fold, *p*‐value 0.082), and Ly6C^+^ high monocytes (1.33‐fold, *p*‐value 0.139), compared to saline‐treated controls (Figure 9B–E). Conversely, HP‐SPION administration induced only a modest increase in numbers of macrophages, DCs, and monocytes (Figure 9D–H). The observed shifts in cell populations reflect the early phase of immunological engagement (24 h), primarily influenced by nanoparticle surface chemistry. However, further investigation into the long‐term adaptive immune response is necessary to fully understand the true biological outcomes.

DS‐SPIONs demonstrated tendential recruitment and interaction with phagocytic myeloid subsets in vivo, aligning with our earlier in vitro findings of efficient internalization by neutrophils, macrophages, monocytes, and DCs. The modest rise in monocyte numbers reflects CCL2‐mediated mobilization from the bone marrow, leading to early inflammatory reprogramming [60]. NK cell enrichment may result from cross‐priming by activated macrophages and a cytokine‐rich milieu.

In contrast, HP‐SPION administration elicited mild changes in splenic immune cell composition, with slight increases in macrophages, DCs, and monocytes observed 24 h post‐injection. This subtle response likely reflects the early timepoint assessed and highlights the tissue‐specific compartmentalization of immune responses. While HP‐SPIONs triggered robust pro‐inflammatory signaling in peripheral compartments such as blood and endothelium, these effects did not translate into broad innate cell expansion in secondary lymphoid organs within this acute phase. Adaptive immune cell analysis revealed minimal perturbations. DS‐SPION administration modestly expanded CD4⁺ T cells by approximately 1.1‐fold, potentially reflecting early immune priming mediated by activated macrophages and neutrophils (Figure 9I). No significant changes were observed in CD8⁺ T or B cell populations in either nanoparticle group (Figure 9J,K). This mild adaptive modulation is consistent with an acute innate‐driven immune engagement phase and supports the potential of DS‐SPIONs as a potent delivery system that induce immune suppressive response that are suitable for regenerative medicine applications.

## 3 Conclusions

Structurally distinct sulfated polysaccharide coatings, specifically DS and HP, on SPIONs differentially modulate immune recognition and downstream functional responses. Both coatings attenuated complement activation and innate immune stimulation in human whole blood. These findings indicate that surface modification with these biopolymers effectively dampens acute innate immune recognition, a critical prerequisite for biocompatible drug delivery systems. Furthermore, their distinct immunomodulatory profiles suggest having divergent therapeutic applications. DS‐SPIONs exhibited preferential uptake by myeloid cells and induced an anti‐inflammatory profile. Conversely, HP‐SPIONs elicited a robust pro‐inflammatory M1‐like macrophage phenotype. These findings underscore the role of surface chemistry and ligand presentation on the nanoparticle surface in immune cell uptake, cytokine response, and endothelial activation that can be ultimately harnessed for various clinical applications. Although the early immunological responses induced by DS‐ or HP‐coated SPIONs suggest their potential utility in fields such as tissue engineering and anticancer therapy, a comprehensive long‐term in vivo assessment within pertinent tissue regeneration/wound healing and cancer models is warranted to fully validate these findings.

## 4 Experimental Section

### 4.1 Materials

Heparin (HP) sodium salt from porcine intestinal mucosa (15 kDa), dextran sulfate (DS) sodium salt from *Leuconostoc spp*. (9‐20 kDa), dopamine (2‐(3,4‐Dihydroxyphenyl) ethylamine hydrochloride, DA), 1‐hydroxybenzotriazole hydrate (HOBt), 1‐ethyl‐3‐(3‐dimethylaminopropyl) carbodiimide hydrochloride (EDC.HCl), 1,1′‐carbonyldiimidazole (CDI), anhydrous dimethyl sulfoxide (DMSO), ferric chloride (FeCl_3_), ferrous chloride (FeCl_2_), sodium hydroxide (NaOH), carbohydrazide (CDH), glycidyl methacrylate (GMA), fluorescein‐5‐thiosemicarbazide (FTSC), fluorescein isothiocyanate isomer I (FITC), and all other reagents and solvents were purchased from Sigma–Aldrich and used as received.

### 4.2 Preparation of Functionalized Biopolymers

HP and DS were chemically modified with DA linkers to facilitate the surface coating of iron oxide nanoparticles. HP and DS was conjugated to DA through carbodiimide coupling chemistry and carbamate‐bond forming reaction, respectively. For HP‐DA synthesis, 1 mmol of HP (per disaccharide unit) was dissolved in 100 mL of deionized water (DIW), followed by the addition of 1 mmol HOBt and 0.8 mmol DA. After stirring to homogeneity, the pH was adjusted to 5.5–6.0, and 0.5 mmol EDC·HCl was added. The reaction proceeded overnight, and the product was purified by stepwise dialysis (MWCO 3.5 kDa) against dilute HCl (pH = 3.5) containing 0.1 M NaCl (3 × 2 L, 24 h), dilute HCl (pH = 3.5) (3 × 2 L, 24 h), and then against DIW (3 × 2 L, 24 h), then lyophilized to yield HP‐DA. For DS‐DA, 1 mmol DS was dissolved in 40 mL anhydrous DMSO under reflux at 90°C. After complete dissolution, the solution was cooled to 40°C, and 1.5 mmol CDI in 10 mL DMSO was added dropwise. Following 5 h activation, 3 mmol DA in 5 mL DMSO was added and stirred overnight at room temperature. The resulting product was diluted with DIW, pH‐adjusted to 5.5‐6, and dialyzed under the same conditions before lyophilization to obtain DS‐DA.

### 4.3 Synthesis of DS‐ and HP‐Coated SPIONs

SPIONs were synthesized by co‐precipitation of FeCl_3_·6H_2_O and FeCl_2_·4H_2_O (2:1 molar ratio) in 25 mL deoxygenated 0.4 M HCl, added dropwise to 250 mL of 0.5 M NaOH at 80°C under nitrogen with vigorous stirring. After 2 h, the resulting black precipitate was collected magnetically and washed repeatedly with ethanol and DIW. For coating, 1 mmol SPIONs were redispersed in 3 mL DIW using probe sonication for 1 h, followed by addition of 1.6 mmol HP‐DA or DS‐DA dissolved in 2 mL DIW. After 30 min water bath sonication, the mixture was refluxed at 90°C (30 min) and further subjected to probe sonication (30 min) to make a homogeneous coating.

### 4.4 Functionalization of HP‐ and DS‐SPIONs for Fluorescein Labelling

For further functionalization, HP‐DA was coupled to carbohydrazide (CDH) via carbodiimide chemistry (1:1 molar ratio), following the protocol described in section 4.2. The product, HP‐DA‐CDH, was purified by dialysis and lyophilized. The degree of hydrazide modification on HP‐DA‐CDH for synthesis of functionalized nanoparticles was measured to be 7.42 mol% with respect to HP disaccharide repeat units through trinitrobenzene sulfonic acid (TNBS) assay (Figure S1F). In the next step, the functionalized nanoparticles were prepared by coating of SPIONs with HP‐DA‐CDH using the same sonication‐heating‐sonication procedure. On the other hand, DS was functionalized by glycidyl methacrylate (GMA) through methacrylation reaction. Briefly, GMA (50 mmol) was added to DS (1 mmol, 1 mg mL^−1^ in PBS pH 7.4) and stirred at room temperature for 48 h, followed by extensive dialysis with a 3.5 kDa MWCO membrane against 0.1 M NaCl solution (6 × 2 L, 48 h), and then against DIW (6 × 2 L, 48 h) to remove unreacted GMA. The lyophilized product, DS‐GMA, exhibited characteristic olefinic proton signals at 5.4 and 5.9 ppm in the ^1^H NMR spectrum (D_2_O), confirming the successful incorporation of vinyl groups. The degree of methacrylation was estimated to be 11% with respect to monosaccharide repeat units as estimated by integrating an anomeric proton 5.15 ppm against vinyl proton at 5.9 ppm (Figure S1G). Accordingly, DS‐coated SPIONs was methacrylated following this same procedure. As the paramagnetic SPIONs are not detectable by NMR, we anticipate that methacrylation of DS‐SPIONs would render similar degree of modification (∼11%) as that of DS polymer used for optimization of this reaction.

### 4.5 Preparation of Fluorescent‐Labeled Nanoparticles

For fluorescent labeling, functionalized HP‐SPIONs reacted with fluorescein isothiocyanate (FITC, 0.075 mmol) dissolved in DMSO and added dropwise to the nanoparticle suspension in a DIW:DMSO mixture (3:7). The reaction mixture was refluxed at 90°C overnight and purified by dialysis against dilute HCl/NaCl, dilute HCl, and DIW. For DS‐SPIONs, FTSC (0.08 mmol) in DMSO was added dropwise to the methacrylated DS‐SPION suspension in DIW:DMSO (1:1), stirred overnight at room temperature, and purified following the protocol reported for HP‐SPIONs. All fluorescent‐tagged formulations were lyophilized and stored protected from light.

### 4.6 Characterization of Nanoparticles

Conjugation of DA to HP and DS was verified by ^1^H‐NMR spectroscopy (JEOL JNM‐ECZR 500 MHz) and quantified using UV–Vis spectroscopy (Shimadzu UV‐3600 Plus UV–vis–NIR spectrophotometer) based on DA absorbance. Hydrodynamic diameter and zeta potential were measured via DLS using a Zetasizer Nano S90 (Malvern Panalytical). The pristine SPIONs were observed by a field emission gun scanning electron microscopy (FESEM) (Carl Zeiss Microscopy GmbH, Germany) operated at a low accelerating voltage (≤1.5 kV). Dried bare SPIONs were placed on carbon adhesive tape attached to standard aluminum stubs and 4 nm platinum‐palladium layer was sputtered to ensure surface conductivity. An in‐lese secondary electron detector was employed to provide high resolution images from the nanoparticles. The coated nanoparticles were investigated by transmission electron microscopy (TEM). For TEM observations, SPIONs were loaded on a TEM copper grids by pipetting a drop of SPIONs suspension and subsequent drying in air. Then, TEM imaging using bright‐field mode was performed in a JEOL JEM‐F200 (Japan) operated at 200 kV. DigitalMicrograph v. 3.52 by Gatan Inc. was used for TEM image processing. Polymer loading on SPIONs was quantified via TGA (SDT Q600, TA Instruments) by heating lyophilized samples under nitrogen from 25 to 1000°C. Fluorescence labeling efficiency and stability were evaluated using (FLS1000 Photoluminescence Spectrometer, Edinburgh Instruments), measuring excitation/emission at (495/517 nm) to confirm successful fluorophore conjugation.

### 4.7 Cytotoxicity Evaluation of Nanoparticles

To evaluate the cytotoxicity of the nanoformulations, mouse embryonic fibroblast (MEF) cells were cultured in Dulbecco's Modified Eagle's Medium (DMEM, high glucose, GlutaMAX supplement) supplemented with 10% FBS (Gibco, Thermo Fisher Scientific, 10500064) and 100 U mL^−1^ penicillin, and 100 µg mL^−1^ streptomycin (Gibco, Thermo Fisher Scientific, 15140122). For the assay, cells were seeded in 96‐well plates at a density of 1 × 10^4^ cells per well and allowed to adhere for 24 h at 37°C and 5% CO_2_.

On the following day, stock solutions of lyophilized DS‐SPIONs and HP‐SPIONs were suspended in cell culture medium and serially diluted. The existing medium on the cells was replaced with 100 µL of fresh medium containing the nanoparticles at final concentrations ranging from 0 to 1 mg mL^−1^. The cells were then incubated for an additional 48 h.

Cell viability was quantified using the Alamar Blue (resazurin) assay (Thermo Fisher Scientific, A50100) according to the manufacturer's protocol. Briefly, after the 48‐h incubation, the nanoparticle‐containing medium was removed, cells were washed once with sterile PBS, and fresh medium containing 10% (v/v) Alamar Blue reagent was added to each well. Plates were incubated for 4 h at 37°C, protected from light. Absorbance was quantified using a Tecan Spark microplate reader (Tecan, Männedorf, Switzerland) at 570 nm, with a reference wavelength of 600 nm used for background correction. Cell viability was expressed as a percentage relative to untreated control cells.

### 4.8 Biocompatibility Evaluation and Thromboinflammatory Response of Nanoparticles

Human lung microvascular endothelial cells (HLMVECs; Cell Applications Inc, San Diego, CA) were cultured until passage five in endothelial growth medium (Sigma‐Aldrich, Steinheim, Germany), as described previously [18]. In brief, cells were seeded in 48‐well cell culture plates (Thermo Fisher Scientific), pre‐coated with 0.1% gelatin solution (Sigma–Aldrich) for 5 min, at 40 000 and 30 000 cells well^−1^ in two separate plates. The plates were used for experiments after 48 and 72 h, respectively. Peripheral blood from six healthy donors was anticoagulated with the thrombin‐specific inhibitor lepirudin (50 µg mL^−1^) to preserve complement activity [61]. Whole blood, 300 µl, was gently mixed with 60 µl of either HP‐/DS‐SPIONs, biopolymers (HP‐DA/ DS‐DA), 0.9% saline, or a positive control, composed of 100 µg mL^−1^ Zymosan A (Sigma–Aldrich), 10 ng mL^−1^ LPS (InvivoGen, San Diego, CA) and 25 µg mL^−1^ TRAP‐6 (Bachem, Bubendorf, Switzerland) in saline and added to the HLMVECs. The coated SPIONs and biopolymers concentration was 200 µg mL^−1^. The 48‐well culture plate was incubated in a cell incubator at 37°C and 5% CO_2_. A 10 µL whole blood sample was isolated after 15 min for flow cytometric analysis of granulocytes and monocytes, and after 60 min for analysis of platelet activation by flow cytometry. The isolated samples were immediately quenched with 20 mM EDTA and CTAD (8 mM trisodium citrate, 1.1 M theophylline, 26 mM adenosine, 14 mM dipyridamole; Greiner Bio‐One, Kremsmünster, Austria) to prevent further activation after isolation. The remaining blood was incubated with HLMVECs for 4 h (37°C, 5% CO_2_), transferred to new tubes, quenched with EDTA (20 mM), and centrifuged (3000 × g, 15 min, 4°C). Plasma was collected and stored at −80°C. Complement split products C3bc, C3bBbP and soluble C5b‐9 terminal complement complex (TCC) were quantified in plasma by enzyme‐linked immunosorbent assays (ELISA), as described previously [62, 63, 64]. PF4 and NAP‐2 levels were quantified with DuoSet ELISA kits (R&D Systems), plasma was diluted 10 000–50 000 times for both analytes. Monocytes, granulocytes, platelets and HLMVECs were stained with fluorochrome‐conjugated antibodies (BD Biosciences, Franklin Lakes, NJ) against CD14/CD11b, CD42a/CD62P/CD63 and MCAM/ICAM‐1/E‐/P‐selectin, respectively, and measured with a CytoFLEX (Beckman Coulter, Brea, CA) flow cytometer.

### 4.9 Mice Acclimatization

Male C57BL/6NTac mice (Taconic) bred at the Comparative Medicine Department at Karolinska University Hospital, Sweden, were maintained in a pathogen‐free and climate‐controlled environment with regulated 12 h light/dark cycles. All mice used for experiments were adults between 2–4 months of age and had access to chow and water *ad libitum*.

### 4.10 Ex Vivo Nanoparticle Uptake by Immune Cells

To assess the cellular uptake of nanoparticles by primary immune cells ex vivo, spleens were harvested from 3‐month‐old male mice following cardiac perfusion with PBS under anesthesia. The spleens were transferred into cold PBS and mechanically minced and passed through a 40 µm cell strainer to obtain a single‐cell suspension. The cells were centrifuged at 350 × g for 5 min at 4°C and incubated with ammonium chloride potassium (ACK) buffer for 10 min at room temperature to lyse the erythrocytes. After washing with PBS, 2 × 10⁵ cells suspended in 100 µL PBS were plated per well in a 96‐well V‐bottom plate. The cells were pelleted and resuspended in RPMI 1640 supplemented with 10% fetal bovine serum (FBS). Then, the cell suspension was transferred to U‐bottom plate and incubated with 0.5 mg mL^−1^ of fluorescently labeled nanoparticles, including DS‐SPIONs‐FTSC, HP‐SPIONs‐FITC, DS‐FTSC, or HP‐FTSC, for 4 h at 37°C in a humidified 5% CO_2_ incubator. Post‐incubation, cells were transferred to a new v‐bottom plate, washed with PBS and used for FACS.

### 4.11 Immunomodulatory Potential of Nanoparticles on Primary Murine Macrophages

To generate bone marrow derived macrophages (BMDMs), femurs were flushed with PBS and the resulting single‐cell suspensions were washed and centrifuged at 350 × g for 5 min at 4°C. The pellet was resuspended in macrophage medium containing Dulbecco's modified Eagle's medium (DMEM; Sigma‐Aldrich, D6046) supplemented with 10% heat‐inactivated fetal bovine serum (FBS; Sigma–Aldrich, F7524), 10 ng mL^−1^ recombinant mouse M‐CSF (R&D Systems, 416‐ML), 2 mM l‐glutamine (Sigma–Aldrich, G7513), 100 U mL^−1^ penicillin, and 100 µg mL^−1^ streptomycin (Sigma–Aldrich, P4458). The cells were cultured in a T175‐cell culture flask (Sarstedt, 83.3912.502) and half the cell medium was replaced on day 4 and fully changed on day 6. The cells were harvested after 7–8 days using trypsin/EDTA solution (Gibco, 25300096), plated for 24 h and subsequently used in experiments.

### 4.12 Reverse Transcription–Quantitative Polymerase Chain Reaction (RT‐qPCR)

For qPCR analysis the cells were plated in 24‐well plates at a density of 2 × 10^5^ cells per well in macrophage medium. The following day, the cells were exposed to different nanoparticles at concentration of 200 µg mL^−1^ dissolved in DMEM (Sigma–Aldrich, D6046) supplemented with 2 mM l‐glutamine (Sigma–Aldrich, G7513), 100 U mL^−1^ penicillin, and 100 µg mL^−1^ streptomycin (Sigma–Aldrich, P4458) for 24 h. The cells were lysed, and total RNA was obtained using the RNeasy mini kit (Qiagen, 74106) with on‐column DNase I digestion using (Qiagen, 79254) according to the manufacturer's instructions. RNA was reverse transcribed to cDNA using iScript kit (BioRad Laboratories, 1708891). Amplifications were conducted in a 384‐well plate using SYBR green (BioRad Laboratories, 1708886) and run in BioRad CFX384 Touch Real‐Time PCR Detection System. Primer specificity was determined by melt curve analysis of each reaction indicating a single peak, and annealing was obtained at approximately 60°C. The primers for the qPCR were obtained from Sigma–Aldrich, Sweden (Table S1).

### 4.13 Flow Cytometric Analysis

Primary murine bone marrow‐derived macrophages (BMDMs) were seeded at 2 × 10⁵ cells well^−1^ in 24‐well plates and incubated with 200 µg mL^−1^ of HP‐/DS‐SPIONs and HP‐/DS‐DA. After 48 h of incubation, cells were detached using trypsin‐EDTA for 10 min at 37°C, neutralized with medium, centrifuged (350 × g, 5 min, 4°C), and processed for flow cytometry. Then, cells were incubated with Live/Dead dye (1:2000) and Fc Block (1:200) for 15 min at 4°C. Surface staining was performed for CD45, CD11b, CD68, CD206 using fluorophore‐conjugated antibodies (1:200) in MACS buffer (PBS with 0.5% BSA and 2 mM EDTA). The fluorophore‐conjugated antibodies used included CD45‐BUV496 (clone 30‐F11, cat# 749889, BD Biosciences), CD11b‐PE‐Cy7 (clone M1/70, cat# 101216, Biolegend), CD86‐BV605 (clone GL‐1, cat# 105037, Biolegend) or CD206‐AF657 (clone C068C2, cat# 141712, Biolegend). After 30 min incubation at 4°C, cells were washed and analyzed on the Cytek Aurora flow cytometer (Cytek Biosciences, USA). Flow cytometric analysis was performed using FlowJo 10.10.0 (BD Biosciences, USA). Gating included singlet and live‐cell selection, CD45⁺ leukocyte identification, and further delineation into macrophages (CD11b⁺) populations. Within CD11b⁺ cells, M1 macrophages (CD86⁺) and M2 macrophages (CD206) were analyzed. Macrophage polarization by nanoparticles was quantified with fluorescence minus one (FMO) control applied to define positive populations and correct for spectral overlap.

### 4.14 Immunomodulatory Potential of Nanoparticles on THP‐1‐Derived Macrophages

The human monocytic cell line THP‐1 was cultured in RPMI‐1640 medium (Gibco, Thermo Fisher Scientific, A1049101) supplemented with 10% FBS (Gibco, Thermo Fisher Scientific, 10500064) and 100 U mL^−1^ penicillin, and 100 µg mL^−1^ streptomycin (Gibco, Thermo Fisher Scientific, 15140122) under standard conditions (37°C, 5% CO_2_). For differentiation into macrophages (M0), THP‐1 cells were seeded in 24‐well plates at a density of 2 × 10⁵ cells mL^−1^ and treated with 100 ng mL^−1^ phorbol 12‐myristate 13‐acetate (PMA) for 72 h, followed by a 24 h resting period in fresh medium. Afterwards, differentiated macrophages were treated with 0.2 mg mL^−1^ nanoparticles for 24 h.

Moreover, pro‐inflammatory polarization was achieved by stimulating differentiated macrophages with 50 ng mL^−1^ LPS and 20 ng mL^−1^ interferon gamma (IFN‐γ) for 48 h, followed by a final 24 h rest period. For anti‐inflammatory polarization cells were treated with 30 ng mL^−1^ IL‐4 for 48 h, followed by a final 24 h rest period. Polarized macrophages were then treated with nanoparticles for 48 h.

Total RNA was extracted using the RNeasy Mini Kit (Qiagen) and reverse transcribed into cDNA using the High‐Capacity cDNA Reverse Transcription Kit (Applied Biosystems, ThermoFisher Scientific). Gene expression levels were quantified by qPCR using TaqMan Fast Advanced Master Mix (Applied Biosystems, ThermoFisher Scientific) on a CFX96 real‐time PCR system (Bio‐Rad). Glyceraldehyde‐3‐phosphate dehydrogenase (GAPDH) served as the reference gene. All primers were sourced from TaqMan Gene Expression Assay (Applied Biosystems, ThermoFisher Scientific) (Table S2).

### 4.15 The Effect of Nanoparticles on Immune Cells In Vivo

Mice were intravenously injected with 100 µL of either nanoparticle (HP‐SPIONs/DS‐SPIONs) or biopolymer (HP‐DA/DS‐DA) at a dose of 8 mg kg^−1^ in sterile saline.

24 h after the injection, mice were anesthetized and perfused transcardially with PBS under deep isoflurane anesthesia. Spleens were collected, minced, and passed through a 40 µm cell strainer. Cells were centrifuged at 350 × g for 5 min at 4°C. Red blood cells were lysed using ACK buffer (5 mL, 10 min, RT), washed with PBS. The cells were pelleted and resuspended in 3 mL PBS, and 120 µL aliquots were plated in 96‐well V‐bottom plates for FACS staining.

4.16

The cell suspensions prepared as described above were blocked with anti‐mouse CD16/CD32 (Mouse BD Fc Block, Biosiences; 1:200) and stained with a live/dead marker (LIVE/DEAD Near‐IR; Dead Cell Stain Kit, Thermo Fisher Scientific;1:2000 in PBS) for 15 min at 4°C. After removal of unbound reagents using MACS buffer, cells were stained with the following fluorochrome‐conjugated antibodies (1:200) targeting surface markers for 30 min at 4°C. CD45‐BUV496 (clone 30‐F11, 749889, BD Biosciences), CD11b‐Percp‐Cy5.5 or CD11b BV650 (clone M1/70, 101228, BioLegend), Ly6C‐AF700 (clone HK1.4, 128024, BioLegend), Ly6G‐AF647 (clone 1A8, 127609, Biolegend), F4/80‐BV421 (clone BM8, 123137, BioLegend), MHCII‐Spark‐UV387 (clone m5/114.15.2, 107669, BioLegend), CD11c‐FITC (clone Bu15, Cat# 337214, BioLegend), NK 1.1‐APC (clone Pk136, cat# 108710, BioLegend), CD3‐Pe‐Cy7 (clone 17A2, cat# 100220, BioLegend) or CD3‐BV510 (clone 17A2, cat# 100233, BioLegend), CD4‐BV785 (clone RM4‐5, cat# 100551, BioLegend) or CD4‐Percp‐Cy5.5 (clone Gk15, cat# 100434, BioLegend), CD8‐PE‐CF594 (clone 53–6.7, cat# 562283, BD Biosciences) or CD8‐PE‐Cy5.5 (clone 53–6.7, cat# 35‐0081‐80, Invitrogen), CD19‐PE‐Dazzle (clone ID3, Cat# 562291, BD Biosciences) or BV650 (clone 6D5, Cat# 115541, BioLegend).

Following a final wash, cells were resuspended in 200 µL MACS buffer and analyzed on a Cytek Aurora spectral flow cytometer (Cytek Biosciences, USA). Flow cytometric analysis was performed using FlowJo 10.10.0 (BD Biosciences, USA). Gating included singlet and live‐cell selection, CD45⁺ leukocyte identification, and further delineation into myeloid (CD11b⁺) and lymphoid (gated on CD11b^−^ and subsequently CD3⁺, CD19⁺, NK1.1) populations. Within CD11b⁺ cells, monocytes (Ly6C⁺), neutrophils (Ly6G⁺), macrophages (F4/80⁺), and DCs (MHCII⁺; CD11c^+^) were analyzed. Lymphoid subsets included CD4⁺, CD8⁺ T cells, CD19⁺ B cells, and NK1.1^+^ NK cells. Nanoparticle uptake was quantified using the FITC channel (B1) to detect fluorescence from conjugated dyes, with FMO control applied to define positive populations and correct for spectral overlap.

### 4.17 Statistical Analysis

Data are presented as mean ± SD. Statistical significance was assessed using GraphPad Prism (v10.3.1). Comparisons between multiple groups were performed using one‐way ANOVA followed by Dunnett's (vs. control) or Tukey's (all pairs) post‐hoc tests. Tests were two‐sided with significance defined as *p* < 0.05. Sample sizes (*n*) were specified in figure legends. Relative gene expression was calculated using the 2^−ΔΔ^ *^C^_T_* method, normalized to housekeeping genes (*Hprt* or GAPDH).

## Ethics Statement

The study was designed and performed according to the ethical guidelines from the declaration of Helsinki and approved by the Ethical Review Board in Linköping, Sweden (Dnr 03–520). Informed written consent was obtained from the blood donors. The experiments were performed in accordance with the Swedish National Board of Laboratory Animals and the European Community Council Directive (86/609/EEC) and the local ethics committee of Stockholm North under the ethical permit 61327‐22.

## Conflicts of Interest

The authors declare no conflicts of interest.

## Supporting information




**Supporting File**: smll72136‐sup‐0001‐SuppMat.docx

## Data Availability

The data that support the findings of this study are available from the corresponding author upon reasonable request.
