# Supplementary material for: Reprogramming Immunogenicity of Iron Oxide Nanoparticles through Sulfated Glycan Presentation
Source: Small. 2026 Feb 1;22(11):e08613. doi: 10.1002/smll.202508613 (PMC12921550; doi:10.1002/smll.202508613)
Supplement: Supplementary file 1 — Supporting File: smll72136‐sup‐0001‐SuppMat.docx [file SMLL-22-e08613-s001.docx]

Supporting Information

Reprogramming Immunogenicity of Iron Oxide Nanoparticles Through Sulfated Glycan Presentation

*Negin Pournoori, Heela Sarlus, Dick J. Sjöström, Rohith Pavan Parvathaneni, Oommen P. Varghese, Vesa P. Hytönen, Robert A. Harris, Per H. Nilsson, Oommen P. Oommen**


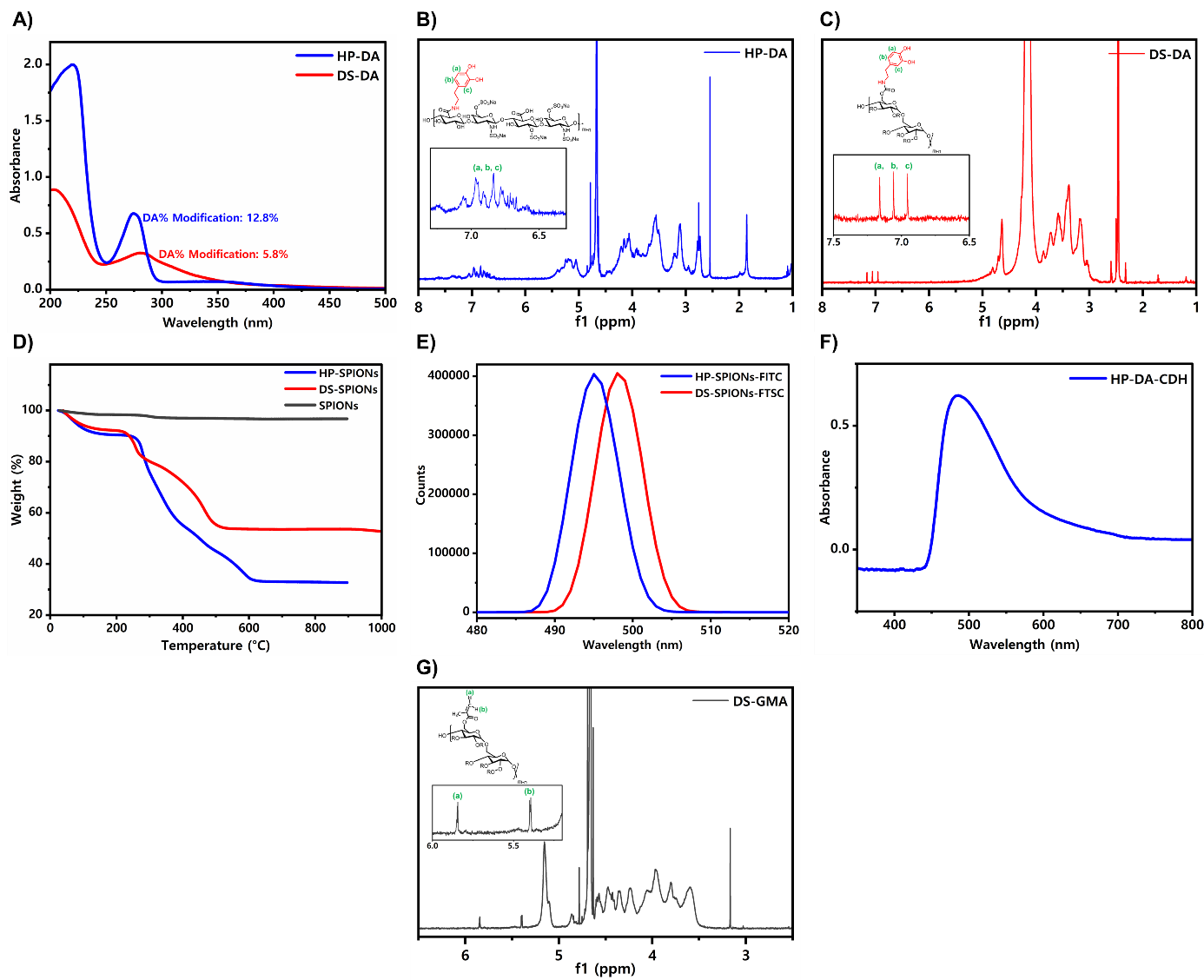


**Figure S1.** (A) UV-Vis spectra of conjugated dopamine in DS-DA and HP-DA dissolved in DIW at 1 mg mL^-1^. ^1^H-NMR (500MHz) spectra of (B) HP-DA and (C) DS-DA recorded in D_2_O and DMSO-d_6_ at 298K, respectively (D) TGA of coated SPIONs (DS- and HP- SPIONs) and uncoated SPIONs (E) Emission spectra of DS-SPIONs-FTSC at 495 nm and HP-SPIONs-FITC at 492 nm (F) UV-Vis spectra of conjugated carbohydrazide in HP-DA-CDH dissolved in 0.1 M sodium bicarbonate buffer (pH 8.5, 0.34 mg mL^-1^). (G) ^1^H-NMR (500MHz) spectra of DS-GMA recorded in D_2_O at 298K.


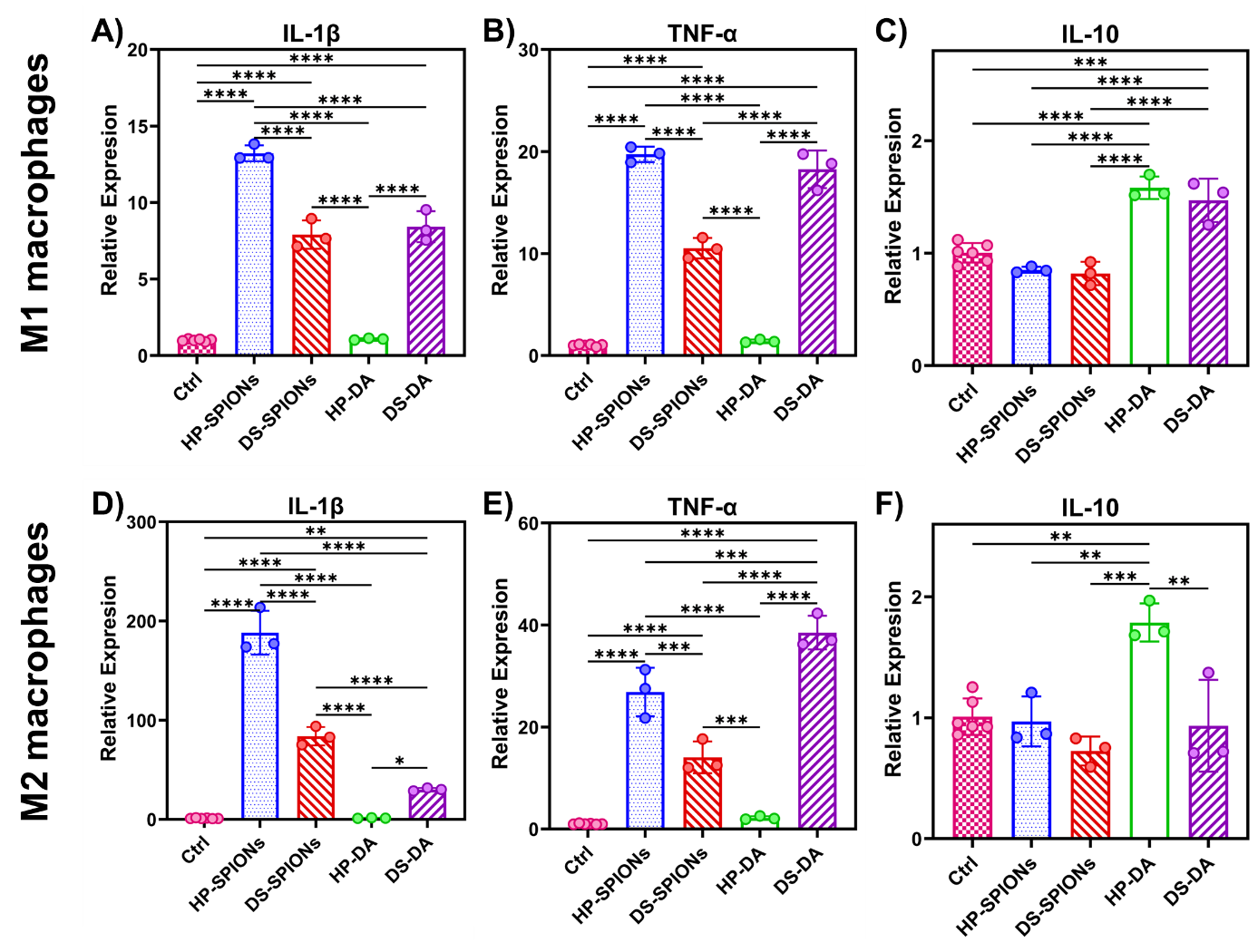


**Figure S2.** **Transcriptional responses of pre-polarized human macrophages to biopolymer-coated SPIONs and their dopamine-functionalized precursors.** Relative expressions of (A) IL-1β, (B) TNF-α, (C) IL-10 in M1 polarized from THP-1 and (D) IL-1β, (E) TNF-α, (F) IL-10 in M2 polarized from THP-1 treated with DS-SPIONs, HP-SPIONs, DS-DA, and HP-DA for 48 h. Gene expression was measured by RT-qPCR and normalized to GAPDH. Data are presented as mean±SD. Statistical analysis was performed using one-way ANOVA with Tukey’s post-hoc test (p<0.05 (*), p<0.01 (**), p<0.001 (***), p<0.0001 (****)).

**Table S1.** List of murine primers and their sequences used for the qPCR experiments.

| **Primer** | **Forward** | **Reverse** |
| --- | --- | --- |
| ***Hprt*** | ACAGCCCCAAAATGGTTAAGG | TCTGGGGACGCAGCAACTGAC |
| ***Il-6*** | TAGTCCTTCCTACCCCAATTTCC | TTGGTCCTTAGCCACTCCTTC |
| ***Il-1β*** | GCAACTGTTCCTGAACTCAACT | ATCTTTTGGGGTCCGTCAACT |
| ***Tnf-α*** | CTGTAGCCCACGTCGTAGC | TTGAGATCCATGCCGTTG |
| ***Nos2*** | GTTCTCAGCCCAACAATACAAGA | GTGGACGGGTCGATGTCAC |
| ***Mrc1*** | TGATTACGAGCAGTGGAAGC | GTTCACCGTAAGCCCAATTT |
| ***Il-4*** | GGTCTCAACCCCCAGCTAGT | GCCGATGATCTCTCTCAAGTGAT |

**Table S2.** List of TaqMan probes used for human gene expression assay.

| **Primer** | **Cat#** |
| --- | --- |
| **GAPDH** | Hs99999905_m1 |
| **IL-1β** | Hs01555410_m1 |
| **TNF-α** | Hs00174128_m1 |
| **NOS2** | Hs01075529_m1 |
| **MRC1** | Hs00267207_m1 |
| **IL-10** | Hs00961622_m1 |
